# Comparison of Family History and SNPs for Predicting Risk of Complex Disease

**DOI:** 10.1371/journal.pgen.1002973

**Published:** 2012-10-11

**Authors:** Chuong B. Do, David A. Hinds, Uta Francke, Nicholas Eriksson

**Affiliations:** 1 23andMe, Mountain View, California, United States of America; 2 Department of Genetics, Stanford University, Stanford, California, United States of America; University of California San Diego and The Scripps Research Institute, United States of America

## Abstract

The clinical utility of family history and genetic tests is generally well understood for simple Mendelian disorders and rare subforms of complex diseases that are directly attributable to highly penetrant genetic variants. However, little is presently known regarding the performance of these methods in situations where disease susceptibility depends on the cumulative contribution of multiple genetic factors of moderate or low penetrance. Using quantitative genetic theory, we develop a model for studying the predictive ability of family history and single nucleotide polymorphism (SNP)–based methods for assessing risk of polygenic disorders. We show that family history is most useful for highly common, heritable conditions (e.g., coronary artery disease), where it explains roughly 20%–30% of disease heritability, on par with the most successful SNP models based on associations discovered to date. In contrast, we find that for diseases of moderate or low frequency (e.g., Crohn disease) family history accounts for less than 4% of disease heritability, substantially lagging behind SNPs in almost all cases. These results indicate that, for a broad range of diseases, already identified SNP associations may be better predictors of risk than their family history–based counterparts, despite the large fraction of missing heritability that remains to be explained. Our model illustrates the difficulty of using either family history or SNPs for standalone disease prediction. On the other hand, we show that, unlike family history, SNP–based tests can reveal extreme likelihood ratios for a relatively large percentage of individuals, thus providing potentially valuable adjunctive evidence in a differential diagnosis.

## Author Summary

In clinical practice, obtaining a detailed family history is often considered the standard-of-care for characterizing the inherited component of an individual's disease risk. Recently, genetic risk assessments based on the cumulative effect of known single nucleotide polymorphism (SNP) disease associations have been proposed as another potentially useful source of information. To date, however, little is known regarding the predictive power of each approach. In this study, we develop models based on quantitative genetic theory to analyze and compare family history and SNP–based models. Our models explain the impact of disease frequency and heritability on performance for each method, and reveal a wide range of scenarios (16 out of the 23 diseases considered) where SNP associations may already be better predictors of risk than family history. Our results confirm the difficulty of obtaining accurate prediction when SNP or family history–based methods are used alone, and they show the benefits of combining information from the two approaches. They also suggest that, in some situations, SNP associations may be potentially useful as supporting evidence alongside other types of clinical information. To our knowledge, this study is the first broad comparison of family history– and SNP–based methods across a wide range of health conditions.

## Introduction

Over the last half decade, genome-wide association studies (GWASs) have revolutionized the conduct of human genetic research. Today, numerous companies offer consumers the opportunity to access their genetic data and provide individuals with personalized interpretations of their data based on genetic associations reported in the literature. Thousands of genetic associations covering hundreds of human diseases and traits have now been discovered [1]; yet, for virtually all complex diseases, the genetic risk factors that have been implicated to date often account for only a small proportion of the total phenotypic variation, even for conditions that are known to be highly heritable [2]–[4].

Recent estimates of the proportion of heritability explained by known susceptibility variants across a survey of ten complex diseases (Alzheimer disease, bipolar disorder, breast cancer, coronary artery disease, Crohn disease, prostate cancer, schizophrenia, systemic lupus erythematosus, type 1 diabetes, and type 2 diabetes) have ranged from 0.4% to 31.2% [5]. These proportions highlight the sobering reality that only a fraction of the genetic contributions to disease have yet been discovered. From a clinical perspective, the problem of missing heritability has spurred substantial concern regarding the practicality of using genetic risk factors in the context of risk prediction. Many of these criticisms focus on the poor predictive value of currently known markers when used in SNP-based risk prediction models, or their limited incremental value when used in conjunction with non-genetic risk factors for disease.

In contrast with genetic associations, family medical history is largely accepted as an important risk factor in clinical diagnosis [6], [7]. Taking a family history can easily be done in a physician's office, over the phone, or from the comfort of home using online web tools [8], [9]. Compared to SNP-based genetic testing, family history risk assessment has the advantage of requiring no specialized equipment and, in its simplest incarnation, can be less expensive than personal genetic testing. Furthermore, family history can be informative of undiscovered genetic factors and shared environmental influences on liability to disease. On the other hand, siblings within a family will generally have the same prediction based on family history, and since half of the genetic variance in a population occurs within families, this poses substantial limits on the degree to which family history can be informative of disease risk.

To date, few direct comparisons of the effectiveness of family history and SNP-based methods for risk prediction across a broad range of diseases have been conducted. A recent study conducted by the Genomic Medicine Institute at Cleveland Clinic compared family history with a commercially available genomic screening service, and found low correlation between the risk estimates given by each approach for three common cancers (breast, colon, and prostate) in a selected population of individuals from a family cancer clinic. These results suggest that the information contributed by family history and current SNP panels may be relatively independent, but do not indicate which method was more likely to be correct in cases where the risk estimates differed [10]. Two other recent studies [11], [12] examined the problem of integrating family history and SNP-based methods for predicting disease risk, but did not specifically quantify the predictive power of each method alone or both methods together.

In this paper, we use a theoretical model to show that the accuracy of family history and SNP-based methods for risk assessment is highly dependent on the particular characteristics of the disease and population group being considered. We find that while family history-based methods are sometimes more effective for highly common diseases, SNP-based risk assessments tend to be more powerful for less common disorders. We use these findings not to argue that SNP-based assessments should replace the use of family history in the clinic, but rather to suggest that SNP-based assessments and family history are best viewed as complementary tools for understanding an individual's predisposition to disease [13].

## Results

The starting point of our analyses is the standard liability threshold model [14], in which the presence or absence of a binary trait is governed by an unobserved continuous phenotype, known as the liability (see Table 1 for a summary of main notation used). Conceptually, the liability (denoted as 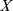) represents the sum total of all the risk factors involved in determining whether or not an individual will develop a particular disease. At the heart of the liability threshold model is the assumption that individuals with liabilities greater than or equal to a fixed threshold 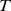 will develop the disease (i.e., cases), whereas individuals with liabilities less than 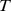 will not develop the disease (i.e., controls).

**Table 1:** Symbols and terminology.

Symbol or Term	Definition
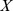	unobserved liability to disease
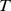	liability threshold, beyond which individual will develop disease
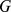	(additive) genetic component of liability
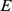	environmental component of liability
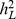	heritability of liability
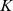	prior probability of developing disease during lifetime
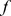	proportion of heritability explained by known SNP associations
area under the curve ( 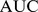 )	probability of a true case receiving a higher predicted risk than a true control
sensitivity	probability of a true case receiving a correct prediction
specificity	probability of a true control receiving a correct prediction
positive predictive value (PPV)	probability of a predicted case receiving a correct prediction
negative predictive value (NPV)	probability of a predicted control receiving a correct prediction
likelihood ratio ( 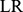 )	ratio of post-test odds to pre-test odds

Usually, the liability is taken to be the sum of two quantities: a genetic component, 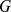, representing the total effect of one's genes on disease susceptibility, and an environmental component, 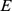, capturing the aggregate of all non-genetic effects influencing the presentation of the disease. We use an additive model of liability, which assumes no contribution due to dominance effects or gene-gene interactions. For polygenic diseases, no single risk factor has a large effect in isolation, and thus (in the absence of gene-environment interactions) 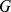 and 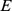 are typically considered to be independent normally distributed random variables.

By convention, we assume 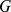 and 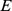 to be independent random variables drawn from zero-mean normal distributions with variances 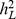 and 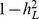, respectively; here, 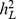 indicates the proportion of variance in 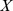 due to additive genetic effects and is known as the *heritability of liability*. Under these assumptions, it follows that 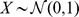 and 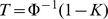, where 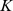 is the disease frequency and 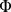 is the cumulative distribution function for a standard normal random variable. Following [5], [11], we interpret 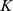 to be the *lifetime morbid risk*, i.e., the probability that an individual will develop a given disease at some point in his or her lifetime.

Here, we consider an extension of the basic liability threshold model to a family-based setting, where the liabilities of related individuals are assumed to have a joint multivariate normal distribution. In this model, the correlations in liability between family members are determined by 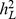 and the degree of genetic relatedness between individuals, assuming no covariance due to shared environmental risk factors. Based on the probabilistic approach for modeling family history proposed in Appendix A of a recent paper by So and colleagues [11], we develop new analytical techniques for estimating the accuracy of family history-based risk prediction models. The details of this procedure are described in Methods.

### Organization of results

**Figure 1:**
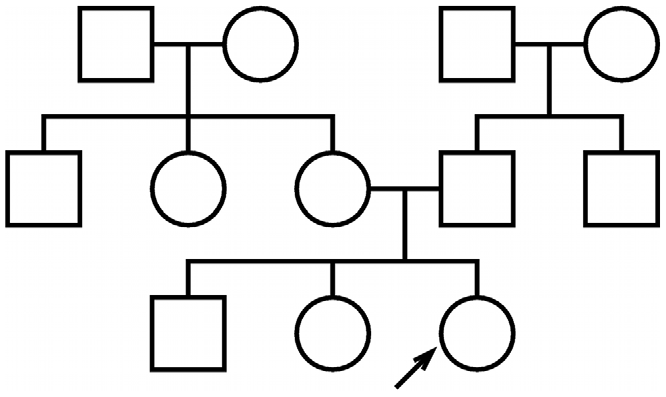
Test pedigree. The test pedigree used throughout this paper, consisting of an extended family with multiple aunts and uncles. An arrow designates one particular individual as the “index individual” (or consultand) whose disease risk we wish to predict.

The power of family history-based risk prediction methods depends substantially on the extent to which an individual's family medical history is known. We analyzed a variety of different test pedigrees, ranging from a simple trio structure to a more complex three-generation family history (see Figure 1 and Figure S1). For the sake of illustration, we focus throughout this paper on the specific three-generation family history in Figure 1; corresponding results for the other pedigrees are generally quite similar (see Figures S2, S3, S4, S5, S6 and Tables S1, S2, S3, S4).

We present the results of our analyses using figures that illustrate the performance of both family history and SNP-based methods under a range of different assumptions regarding the characteristics of the polygenic disease being modeled. Each figure is structured as a rectangular array of plots, organized to illustrate the dependence of predictive accuracy on the heritability of liability (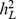) and disease frequency (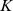). For consistency, rows in each figure correspond to varying choices of heritability (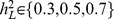), and columns correspond to varying choices of disease frequency (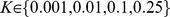). Array cells correspond to particular combinations of heritability and frequency.

We depict the performance of family history based on two different models. First, we consider a *complete* family history-based model (solid red line) that assesses an index individual's risk based on the exact pattern of disease occurrence in his or her relatives. Second, we consider a *restricted* family history-based model (dotted red line) that only takes into account the number of first-degree relatives of the index individual who have the disease, bucketed into three categories (either 0, 1, or 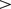
1). Results for the complete model are appropriately interpreted as a theoretical “best-case” analysis for family history: among all predictive models that rely only on the pattern of disease occurrence within the family, the complete model achieves the highest accuracy possible under various assumptions. The more moderate levels of performance achieved by the restricted model, however, are likely more representative of what is actually achieved in clinical practice (see, e.g., the incorporation of family history in the Gail breast cancer model [15]).

We depict the performance of SNP-based risk assessment assuming that only a proportion (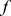) of the genetic factors underlying disease liability are accounted for by known disease associations. Our performance estimates for SNP-based models assume a normal distribution of genetic liability among cases and controls [16], [17], and do not take into account either the disease status or genetic factors of any relative.

### Risk stratification

**Figure 2:**
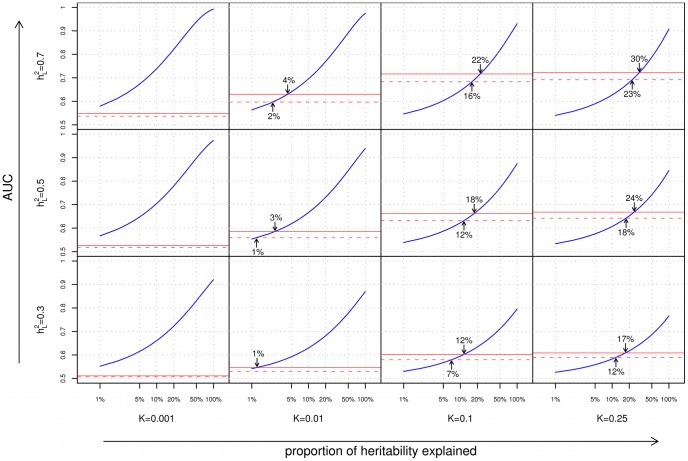
Area under the curve (AUC) plots. Each cell of the 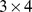 grid corresponds to a different combination of disease characteristics: rows correspond to differing heritabilities (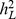) and columns correspond to differing frequencies (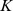). Within each cell, the subplots compare the AUC when using a complete family history model that accounts for the disease status of every individual in the pedigree (solid red line), a restricted family history model that only considers the number (0, 1, or 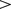
1) of affected first-degree relatives of the index individual (dashed red line), or genetic risk factors for the index individual only (blue line). For each subplot, the horizontal axis indicates the proportion (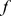) of heritability explained by known SNP associations, and the vertical axis indicates the AUC. Arrows indicate points of equivalence—values of 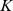 and 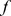 for which family history and SNP-based methods give the same AUC.

We begin our analyses with a comparison of the ability of family history and SNP-based models for stratifying individuals according to risk. In Figure 2, we consider a common measure of discriminative accuracy for risk stratification: the area under the receiver operating characteristic (ROC) curve, also known as the AUC. In each plot, the horizontal axis corresponds to the proportion (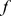) of the additive genetic liability explained by known SNP associations, and the vertical axis indicates the AUC, ranging between 0.5 (random guessing) and 1 (perfect discrimination). ROC curves for the various methods tested are shown in Figure S4.

From Figure 2, we can make a number of observations regarding the relative performance of family history and SNP-based methods for risk stratification:

#### Dependence on heritability and disease frequency

As one might expect, the accuracies of both family history and SNP-based models increase with increasing heritability of the underlying disease. However, the response of each approach to varying disease frequency differs substantially. Family history fares well for highly common conditions, and worse for rarer diseases; this is to be expected since for an uncommon disease, the vast majority of index individuals in a population will have no affected relatives in their pedigrees. Genetic risk prediction models, on the other hand, show the opposite trend; as disease frequency decreases, SNP-based risk prediction models tend to exhibit better discriminative performance. This is consistent with the previously reported observation that the maximum AUC for a SNP-based risk prediction model increases with decreasing disease prevalence [16].

#### Complete versus restricted family history

The discriminative accuracies of the complete and restricted family history models are generally close, with the advantage of the former over the latter being most pronounced for large pedigrees where the restricted model fails to look beyond the closest relatives, or where the limited set of family history categories considered (i.e., 0, 1, or 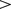
1 first-degree relative) prevents the restricted model from distinguishing between higher numbers of first-degree relatives with the trait (see, e.g., the disparity between complete and restricted family history for the pedigree in Figure S1B, as shown in Figure S2B). In all cases, however, discriminative accuracy for even the optimistic complete family history model peaks at 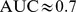 for higher heritability (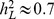) diseases and at 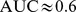 for lower heritability (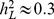) diseases.

#### Comparison of family history and SNP–based models

Finally, of particular note are the locations on the various graphs where the family history and SNP-based risk prediction curves meet, corresponding to the minimum proportion of heritability that must be explained for a SNP-based risk prediction model to match family history in discriminative accuracy. The locations of these intersection points vary widely as a function of both disease frequency and heritability. Family history is most effective for diseases of high frequency and high heritability, accounting for as much as 20–30% of the heritability of the disease, thus meeting or exceeding the best SNP-based models based on GWAS associations to date. But conversely, for complex diseases of low frequency and/or low heritability, SNP-based models surpass family history even when very little of the total genetic variance has been explained. Specifically, for diseases with 1% frequency, the crossover point occurs at less than 4% of the genetic variance explained, which is well within the proportion of heritability explained by known genetic variants for a wide range of diseases [5].

**Figure 3:**
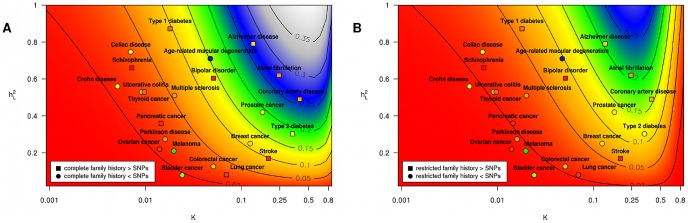
Proportion of heritability explained. Subpanels (A) and (B) contain contour plots showing the proportion of heritability explained (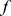) by a complete family history model and a restricted family history model, respectively. Horizontal and vertical axes correspond to varying disease frequency (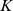) and heritability (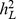). Lines in each subplot depict the level curves of 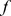, i.e., the combinations of 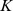 and 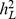 for which the proportion of heritability explained by family history is constant. SNP-based risk models for specific diseases are illustrated by circles (when the SNP-based model outperforms family history) and squares (when family history outperforms the SNP-based model). The circle or square for each SNP-based model has been colored to indicate the estimated proportion of heritability explained by SNPs, using the same color scheme as the contour plot (e.g., blue indicates 25–30% of heritability explained whereas red indicates 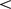
5% of heritability explained). Note that the performance of SNP-based models shown here reflects only currently known genetic factors for European populations and will change as more associations are discovered.

This last point is seen most clearly in the context of real diseases, as shown in Table 2 and Figure 3. We show for a variety of health conditions (including age-related macular degeneration, Alzheimer disease, bipolar disorder, bladder cancer, breast cancer, celiac disease, colorectal cancer, coronary artery disease, Crohn disease, lung cancer, melanoma, multiple sclerosis, ovarian cancer, pancreatic cancer, Parkinson disease, prostate cancer, schizophrenia, stroke, thyroid cancer, type 1 diabetes, type 2 diabetes, and ulcerative colitis) the predictive accuracies achieved by complete and restricted family history risk models. We translate these accuracies into estimated proportions of heritability explained by family history, which we then compare against a variety of SNP-based models based on known associations from an online catalog of published GWAS associations maintained by the National Human Genome Research Institute.

**Table 2:** Performance comparison summary for test pedigree in Figure 1.

			AUC			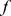		
Disease	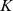	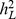	complete	restricted	SNPs	complete	restricted	SNPs
Coronary artery disease	0.402	0.49	0.665	0.627	0.584	26.3%	15.8%	6.9%
**Type 2 diabetes**	**0.339**	**0.30**	**0.610**	**0.587**	**0.592**	**18.6%**	**11.7%**	**13.0%**
Atrial fibrillation	0.245	0.62	0.701	0.673	0.593	27.8%	20.6%	6.1%
Stroke	0.190	0.17	0.564	0.552	0.528	9.8%	6.3%	1.9%
***Prostate cancer***	***0.165***	***0.42***	***0.606***	***0.583***	***0.614***	***10.2%***	***6.3%***	***11.9%***
Alzheimer disease	0.132	0.79	0.743	0.712	0.648	27.1%	20.6%	10.0%
***Breast cancer***	***0.123***	***0.25***	***0.560***	***0.544***	***0.586***	***5.2%***	***2.8%***	***10.5%***
**Lung cancer**	**0.069**	**0.08**	**0.527**	**0.519**	**0.525**	**2.6%**	**1.3%**	**2.3%**
Bipolar disorder	0.051	0.60	0.675	0.637	0.550	13.9%	8.5%	1.1%
***Colorectal cancer***	***0.051***	***0.13***	***0.538***	***0.526***	***0.564***	***3.1%***	***1.4%***	***8.6%***
***Age-related macular degeneration***	***0.047***	***0.71***	***0.700***	***0.660***	***0.758***	***15.2%***	***9.7%***	***25.9%***
***Bladder cancer***	***0.024***	***0.08***	***0.517***	***0.511***	***0.577***	***0.8%***	***0.3%***	***16.2%***
***Multiple sclerosis***	***0.020***	***0.51***	***0.615***	***0.582***	***0.622***	***5.4%***	***2.7%***	***6.1%***
***Melanoma***	***0.020***	***0.21***	***0.544***	***0.528***	***0.640***	***1.9%***	***0.8%***	***19.7%***
Type 1 diabetes	0.018	0.87	0.700	0.660	0.638	9.6%	6.1%	4.5%
***Parkinson disease***	***0.016***	***0.27***	***0.553***	***0.534***	***0.592***	***2.0%***	***0.8%***	***6.0%***
**Pancreatic cancer**	**0.015**	**0.36**	**0.569**	**0.545**	**0.557**	**2.5%**	**1.1%**	**1.7%**
***Ovarian cancer***	***0.014***	***0.22***	***0.520***	***0.513***	***0.548***	***0.3%***	***0.1%***	***2.0%***
***Thyroid cancer***	***0.010***	***0.53***	***0.591***	***0.563***	***0.614***	***2.7%***	***1.3%***	***4.3%***
***Ulcerative colitis***	***0.009***	***0.53***	***0.588***	***0.561***	***0.666***	***2.5%***	***1.2%***	***9.2%***
Schizophrenia	0.007	0.66	0.607	0.578	0.540	2.8%	1.5%	0.4%
***Celiac disease***	***0.007***	***0.75***	***0.624***	***0.594***	***0.733***	***3.4%***	***1.9%***	***12.6%***
***Crohn disease***	***0.005***	***0.56***	***0.573***	***0.551***	***0.717***	***1.4%***	***0.7%***	***13.5%***

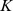
 denotes the lifetime morbid risk, and 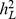 denotes the heritability of liability. The next three columns provide discriminative accuracies (measured in terms of 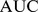) for complete family history, restricted family history, and currently known SNP associations. The last three columns show the corresponding estimated proportion of heritability explained by each model (for family history models, this is taken to be the proportion of heritability that a SNP-based model would need to explain in order to obtain the given 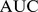). Bolded, italicized rows indicate diseases for which current SNP-based models outperform the complete family history model; bolded, non-italicized rows indicate diseases for which current SNP-based models outperform the restricted but not the complete model. Diseases were selected based on availability of disease frequency and heritability estimates; references for values of 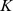, 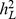, and 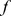 (for SNP-based models) are provided in Text S2 and Table 3. Note that the performance of SNP-based models shown here reflects only currently known genetic factors for European populations and will change as more associations are discovered.

Obtaining accurate epidemiological parameters for each disease is difficult in practice. Heritability estimates, in particular, vary widely, and depending on the methodology used, estimates of the proportion of heritability explained by known genetic factors also differ. In Materials and Methods, we describe a conservative procedure for estimating 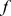 that incorporates a number of corrections to avoid overstating the accuracy achievable with currently known associations. We note that accuracy estimates may vary depending on the specific criteria used for SNP selection, so the numbers provided are meant to suggest general trends in performance across diseases, rather than providing precise benchmarks for the models used by existing commercial personal genomic screens. The above caveats not withstanding, for the 23 conditions included in the table and figure, current SNP-based risk models outperform complete family history for 13 out of 23 conditions and outperform restricted family history for 16 out of 23 conditions, with the magnitude of the differences in performance greatest for diseases of low frequency.

### Standalone prediction

On a population level, risk prediction models may be useful as tools for stratifying individuals based on risk, so as to optimize the allocation of resources for disease prevention programs. On an individual level, however, family history and SNP-based models provide only limited power for predicting disease outcomes when used in isolation. This can be seen most clearly in terms of positive predictive value (PPV), which, for a dichotomous test, is the probability that a positive result is actually indicative of disease.

In general, the risk estimates generated by a family history or SNP-based model are not direct predictions of disease status; however, for any threshold 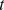, one can define a classification algorithm that predicts an individual will develop the disease only when his or her estimated risk is greater than 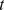. By varying the choice of 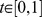, we can obtain a family of dichotomous prediction algorithms that are capable of achieving varying combinations of sensitivity and specificity. Figure S5 shows the PPV obtained at different levels of sensitivity. A corresponding plot of negative predictive values (NPVs) is provided in Figure S6.

For polygenic diseases of low frequency, the predictive value of a classifier based on either family history or SNPs will be extremely poor. Even a risk model that accounts for half the variance in total liability (e.g., a SNP-based risk model that accounts for 100% of the heritability for a disease with 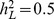, or two-thirds of the heritability for a disease with 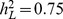) will still have very limited power to accurately identify cases when 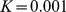; such a classifier, when tuned to obtain a sensitivity of only 10%, would be correct on only one out of every five positive predictions, and thus would have a false positive rate of 80%. At the same level of sensitivity, a family history-based method would have essentially zero probability of a positive prediction being correct.

### Differential diagnosis

**Figure 4:**
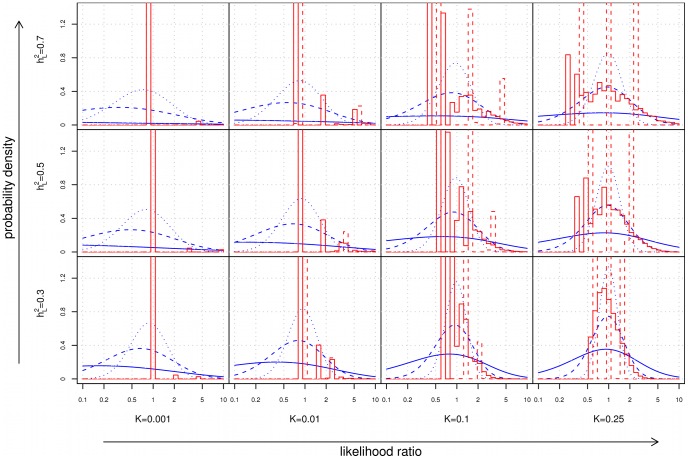
Distribution of likelihood ratios (LRs). The subplots show density histograms for the distribution of LRs achieved by complete (solid red line) and restricted (dotted red line) family history models, and probability density functions for the distribution of LRs achieved by genetic factors accounting for either 10% (dotted blue line), 30% (dashed blue line), or 100% (solid blue line) of the heritability of the disease. As a technical aside, since all plots are shown on a logarithmic scale, the density histograms and density functions shown here were derived for 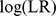 rather than 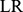 itself (see Text S1).

Based on the results of the last section, one might conclude that for polygenic diseases, the usefulness of family history and SNP-based tests is primarily limited to diseases that are extremely common and highly heritable. Consider, however, a different application: diagnosis of a disease when other risk factors have already led a physician to suspect that a particular condition may be present. In this scenario, the quantity of relevance is not the predictive value of the test in isolation, but rather the strength of the evidence provided by the test when combined with observed symptoms, clinical signs, and other risk factors for disease. Numerically, the strength of evidence is expressed in terms of a *likelihood ratio* (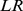), the multiplicative factor by which the odds of having a disease change after seeing the results of the test, analogous to the concept of “odds ratio” in an epidemiological study. Figure 4 shows the distribution of LRs in a population for family history and SNP-based risk models.

As disease frequency decreases, the range of LRs observed for both family history and SNP-based models increases. However, the proportion of individuals receiving extreme (i.e., very large or very small) LRs decreases dramatically for family history, a consequence of the fact that the probability of having a positive family history also falls. For instance, using our test pedigree, for a relatively common disease of high heritability (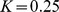, 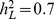), roughly 2.6% of the population has at least 5× increased or decreased odds according to a complete family history model; at 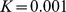, this fraction falls to 0.4%.

In contrast, the availability of SNP-based evidence does not depend on observing disease in relatives, so extreme LRs are in fact more likely. For the example above, a SNP-based model explaining 30% of the heritability of the disease would assign at least 5× increased or decreased odds to 8.5% of the population at 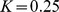, but this increases to 49.2% at 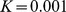. Of the individuals in this 49.2%, the bulk (roughly 92%) receive test results indicating that they are at decreased risk.

### Combining family history and SNP–based models

Although family history and SNPs are often considered alternative tools for risk assessment, methods that integrate information from both family history and SNP-based risk assessments can be more informative than either approach individually [11], [12]. One challenge in building combined models is the potential non-independence of family history and SNP-based risk. For a complex disease, family history will generally capture a portion of the variability in genetic liability explained by SNPs; in Methods, we describe an extension of our accuracy estimation approach to deal with combined family history and SNP-based models that accounts for non-independence without the need to resort to heuristic arguments. Using this extension, we find that in some cases, the proportion of overlap in explained variance due to non-independence can be non-trivial (see Table S3). For example, family history and SNP-based methods separately achieve AUCs of roughly 0.70 and 0.76 for predicting risk of age-related macular degeneration, corresponding to 15% and 26% of the heritability of the disease, respectively. Combining the two methods yields an estimated AUC of roughly 0.80, corresponding to 36% of the heritability of the disease, implying that effectively 5% of the variability in total liability (equivalently, roughly one-third of the variance explained by family history or roughly one-fifth of the variance explained by currently known SNP associations) is shared between family history and SNPs. For diseases where a lower proportion of heritability has been explained, the effect of non-independence is smaller.

## Discussion

We used quantitative genetic theory to compare the predictive accuracy of family history and SNP-based approaches for predicting risk of polygenic diseases. We focused on three key areas: risk stratification, standalone prediction, and differential diagnosis. In each area, we investigated a wide range of theoretical and actual diseases and identified major trends in performance for both family history and SNP-based models.

### Interpretation

In terms of risk stratification, we found that family history is most effective for diseases of high frequency and heritability, such as atrial fibrillation, Alzheimer disease, or coronary artery disease, in each case explaining 20–30% of the genetic variance. The predictive power of family history, however, diminishes quickly with decreasing disease frequency, such that family history explains less than 4% of the heritability for less frequent diseases (e.g., celiac, schizophrenia, or Parkinson disease). In contrast, SNP-based models do not show the same dependence on disease frequency, and for the majority of the diseases we investigate, SNP models based on currently known associations perform as well or better than family history (see Table 2).

For both types of models, high predictive value is extremely difficult to achieve for standalone prediction of disease, especially for less common conditions. This observation should be unsurprising to those familiar with the difficulty of achieving high positive predictive values for rare diseases: for an uncommon condition such as Crohn disease, even a diagnostic test that is able to identify an individual as having a 100-fold increased odds of having the disease only raises the post-test probability to roughly one in three. The fraction of individuals with high estimated risk is also very small; a genetic classifier explaining 100% of the heritability for Crohn disease would classify less than 0.03% of all individuals as having greater than 50% disease risk (see Text S1).

In practice, there do exist some exceptional circumstances where meaningful predictive value may be achievable from a standalone prediction tool. For instance, high-penetrance Mendelian mutations (which are explicitly excluded from our analysis, but see [18]) are commonly used for diagnosis of asymptomatic individuals, or for assessing the risk that couples will pass a specific inherited disorder on to their progeny. Also, for highly common disorders, the odds ratios needed to obtain clinically significant risks of disease are not particularly large; for instance, germline mutations in *BRCA1* or *BRCA2* provide only 7-fold to 9-fold increases in the odds of a woman developing breast cancer, yet result in post-test risks ranging from 49% to 57% [19] due to the high prior probability of the disease (roughly one in eight). Nonetheless, the results of this paper suggest that for most diseases of low or moderate frequency, in the absence of known strongly penetrant mutations, obtaining high predictive value using SNP-based risk models will likely remain challenging, even if additional SNPs are discovered that explain more of the heritability of disease [20].

We note that the performance considerations underlying the usefulness of a risk prediction algorithm can be very different at the population level compared to the individual level. In a large population, mild increases in discriminative accuracy (as measured using the AUC) may have important consequences on the effectiveness of public health initiatives that use risk stratification to efficiently allocate resources for disease screening and prevention. The analysis of clinical utility for a risk stratification algorithm involves many complicated factors beyond the predictive performance of the algorithm, and we do not address these issues here [21] (though see [11]).

In the context of differential diagnosis, we found that SNP-based models consistently produced wider distributions of likelihood ratios than family history. Although these differences are most dramatic for low frequency diseases, the absolute differences in risk remain low; for instance, individuals with a 10× increase in odds for a disease with 0.01% general population frequency will still not have the disease roughly 99.9% of the time.

However, when a physician contemplates the likelihood of a particular disease in the context of a differential diagnosis, the “effective pre-test risk” is actually much larger than the general population risk 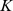, since other non-genetic factors may already be present which raise the odds of the disease. Consider, for example, Crohn disease where we have estimated that current SNP-models explain approximately 13.5% of the heritability. In an unselected population, roughly 8.2% of all individuals would be identified as being at 5× increased or decreased odds of the disease (or 1.7% at 10× increased or decreased odds). For a patient for whom a diagnosis is already suspected on the basis of clinical symptoms (e.g., abdominal pain, diarrhea, fever, rectal bleeding, elevated white blood cell counts), the information provided by a SNP-based test may help to support or weaken this hypothesis.

Note that in this type of setting, the utility of family history and SNP-based tests differs considerably. The extreme likelihood ratios provided by SNP-based tests, when combined with non-genetic factors, may contribute valuable adjunctive evidence to a diagnostic work-up. For family history, however, the low probability of extreme likelihood ratios means that few individuals will have useful information that can meaningfully contribute to the diagnosis of an uncommon disease.

Finally, we note that our results, which suggest that SNP-based tests will often yield extreme LRs indicating decreased risk of disease, differ qualitatively from the conclusions reached in a recent study by Roberts and colleagues [22], who argued that the negative test results from a sequencing-based genetic test would “in general, not be very informative, because the risk of developing [… disease] in those who test negative will still be, at minimum, 50 to 80% of that in the general population”. We attribute the above difference to the fact that the latter study assumed a population genetic model in which the minimum risk for any individual in the population was constrained to be 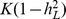.

### Limitations

Our analyses rely on a simple liability threshold model of family history that exclude a number of factors affecting risk estimates:

#### Highly penetrant mutations

Multifactorial models assume that liability consists of the combined action of many independent variants with small additive effects. This excludes highly penetrant mutations with well-characterized inheritance patterns, such as *BRCA1*/*BRCA2* mutations in breast cancer. In such situations where the distributions of genetic liability deviate significantly from normality, extensions of the liability threshold model to incorporate a major locus may provide improved accuracy estimates [23].

#### Age-of-onset

For a disease with generally late age-of-onset, observing a relative with early-onset disease provides stronger evidence of a significant role for genetic factors than if the relative had typical onset. Early age-of-onset is often indicative of the involvement of familial disease due to high-penetrance mutations.

#### Non-additive effects, shared environment, and population structure

The liability threshold model excludes non-additive genetic effects resulting from gene-gene or gene-environment interactions, covariance in liability due to common shared environment between family members, and shared genetic covariance arising from population structure (e.g., consanguinity). In principle, if one could accurately characterize the proportion of variance arising from each of these sources, then it would be straightforward to include these components in our liability threshold model; however, obtaining stable estimates of these parameters for individuals of varying relationships is difficult.

The effects of each of the above factors on our results are arguably limited, and are thus unlikely to substantially influence population-wide measures of accuracy such as AUC. For example, inherited *BRCA1*/*BRCA2* mutations have been estimated to be present in approximately 5% of all breast cases [24] and approximately 12% of all invasive ovarian cancer cases in unselected populations [25]. Depending on the disease, early-onset cases typically account for only a small percentage of the total disease burden (e.g., 6–7% for Alzheimer disease [26]). Drawing from empirical studies, Hill and colleagues have argued that for most complex traits, additive variance accounts for at least one-half (and often close to 100%) of the total genetic variance [27].

The importance of shared environment varies by condition but across different cancers has been estimated to account for no more than 17% of the total variance in liability [28]. In Methods, we describe a further extension of our liability threshold model for upper-bounding the contribution of shared environment to accuracy; these results, which are presented in Tables S2 and S4, suggest that increases in the performance of family history-based models due to shared environment factors are unlikely to significantly change the broad patterns of performance identified in this paper.

Additional considerations affecting the performance estimates in this paper include:

#### Use of lifetime risk for disease frequency

Our measure of disease frequency is the lifetime morbid risk, the probability that an individual will develop a disease in his or her lifetime. Thus, our model of family history relies not on the known disease status of individuals in the pedigree at a single point in time, but rather the pattern of disease occurrence based on the entire lifetime of each individual. While this may be reasonable for pedigrees in which all relatives are suitably old (and explains why we focused on pedigees where the index individual belonged to the youngest generation), it implies that the discriminative accuracies we estimate for family history are likely to be somewhat higher than those achievable in most situations.

#### Recall biases and limits of clinical interpretation

Here we assume all family histories are complete and error free. In practice, the implementability of such a diagnostic model may be hampered by recall biases resulting from an individual's incomplete knowledge of his or her family history, or the inability of a clinician to correctly make fine-grained distinctions in risk based on subtle differences in the pattern of disease occurrence in a family. The net effect of this would again be an inflation of the estimated discriminative performance for family history-based risk prediction.

#### Estimates of heritability

The quantitative genetic model used in this paper depends largely on accurate estimates for disease heritability. Measurements of heritability depend strongly on the particulars of the population in a study; the extent to which the heritability measured in one particular population will generalize to other populations with different ethnic composition, age distribution, geographic location, or other environmental context is unclear [29]–[31]. Furthermore, estimates from twin studies tend to have extremely large standard errors and can be biased when interactions are not properly taken into account [32].

We have attempted to ameliorate the above issues by examining predictive performance across a wide range of conditions and limiting our conclusions to those which appear to generalize well across different values of heritability. It should also be noted that errors in heritability estimates have similar direction of effect for both our family history and SNP-based models. Thus, though they may affect the absolute estimates of accuracy, they will not change the broad results.

Finally, it is worth considering the applicability of our results in general populations. The SNP-based models examined in this paper were restricted to associations derived in European populations. For non-European individuals or individuals of mixed ancestry, it is unclear to what extent the accuracy estimates presented here for SNP-based models will apply due to differences in allele frequencies, linkage disequilibrium between tagging SNPs and true causal variants, and odds ratios across populations. Furthermore, ascertainment biases in genetic studies (e.g., due to recruitment of only severe cases) or differences in phenotype definition (e.g., between study inclusion criteria and more commonly used clinical criteria) can distort estimates of the effectiveness of a SNP-based model in real populations. Similarly, the family history-based accuracy estimates may also vary depending on the extent to which disease frequencies and heritabilities are similar across populations.

### Significance

Over the last decade, family history tools have seen growing adoption with the development of public health efforts focused on prevention [7], [33], [34]. In the United States, the Centers for Disease Control and Prevention (CDC) have developed Family Healthware, an interactive online tool for personalized familial risk assessments for six common chronic diseases (coronary heart disease, stroke, diabetes, colorectal cancer, breast cancer, and ovarian cancer) [8], [9], [35]. In collaboration with the United States Surgeon General and other federal agencies, the CDC's Office of Public Health Genomics has also been involved in the deployment of “My Family Health Portrait,” a web-based tool to help individuals collect and organize their family health history information.

The relative acceptance of family history methods contrasts with the mixed reception of genetic testing in recent years. In some cases, the lack of disease-specific randomized clinical trials assessing clinical utility in terms of improved health outcomes has been cited as a reason for not performing genetic tests [36]. While the demonstration of clinical utility for SNP-based risk assessments is still ongoing, it is nonetheless worth noting that many of the same challenges still exist for family history-based tools. Few studies have sought to validate the accuracy of family history-based models for predicting clinical outcomes in unselected populations, and limited scientific evidence exists regarding the effectiveness of family history-based messaging for motivating behavioral changes for disease prevention [13], [37], [38]. It has been previously suggested that an AUC of 0.75 to 0.8 provides a decent rule-of-thumb for determining when a test may be useful for population screening to identify individuals at increased risk for a disease [39]. Based on such criteria, family history-based stratification would be unlikely to be useful for screening except under the best-case circumstances of extremely common, heritable disorders.

We re-emphasize that we have focused on polygenic diseases where no single risk factor has a substantial individual contribution to liability. Our conclusions, therefore, are not necessarily applicable in situations where a causal mutation is known and easily typed (where SNP-based tests have an advantage) or situations involving unknown highly penetrant genetic risk factors (where family history has an advantage). The extent to which human diseases are governed by rare variants of large effect versus common variants of moderate or low effect is a subject of substantial debate in human genetics [40]. It is worth recalling, however, that for many complex diseases, the majority of disease burden is idiopathic, i.e., the contribution of known high penetrance mutations to disease susceptibility is very small.

As the cost of obtaining genetic information continues to decrease, we believe that access to genetic information will become increasingly common. The implications of widespread genetic testing for public health are still unclear, and the challenge of how best to incorporate adjunctive genetic information into clinical decision-making is far from resolved. But in some circumstances, genetically-defined disease predispositions known from birth may be one of the few clues that an individual will have for anticipating and preventing future morbidity.

Broadly speaking, the personalization of healthcare will require better approaches for integrating different sources of knowledge and for interpreting and communicating the resulting information. As shown in this paper, there exist distinct regimes of disease frequency where family history and SNP-based tests each have an advantage. More importantly, however, methods that combine the results of family history and SNP-based risk assessments can be more informative than either one individually.

In this sense, comparisons of family history and SNP-based methods aimed at declaring one method categorically superior to the other create a false dichotomy: in general, there is no need to choose between family history and genetic risk profiling. An understanding of both types of information would allow us to obtain a better picture of an individual's potential future health. To ignore the potential impact of genetic information on public health, while choosing to rely only on traditional risk factors such as family history, will become increasingly untenable as our understanding of genetics grows.

## Methods

### Modeling family structure

We consider an extension of the liability threshold model to account for the correlations in genetic liability arising from family structure. This extension follows immediately from the original work of Falconer [14], and its application to modeling family history was more recently considered by So and colleagues [11]. The text in this subsection summarizes the general modeling approach described in Appendix A of the latter paper.

For a group of 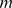 genetically related individuals, the liability of each individual in the group consists of additive genetic and environmental contributions, 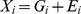. Due to genetic sharing between individuals in a family, however, one would expect the various genetic contributions for different family members to be correlated to varying degrees. A natural model of genetic covariance is to assume that 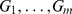 have a joint multivariate normal distribution with zero mean and covariance matrix 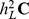, where 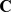 is the 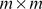 matrix of genetic relationship coefficients for each pair of individuals in the family (e.g., 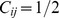 for parent-children or full sibling relationships). Similarly, 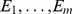 may be treated as jointly multivariate normal with zero mean and scaled identity covariance matrix 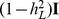, assuming no shared environmental contributions to liability.

For notational convenience, suppose that 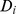 is an indicator variable that takes the value 1 whenever 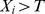 and 0 otherwise. Conceptually, the family history pattern of the first individual in the group (whom we denote as the index individual) can be thought of as the 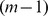-dimensional vector of disease statuses 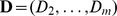 for each of his relatives.

As a simple example, consider the pattern of disease liabilities in a small family consisting of an index individual and his two parents. Letting 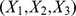 denote the total liabilities of the index individual, his father, and his mother, respectively, and similarly for 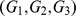 and 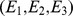, we have
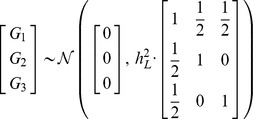
and
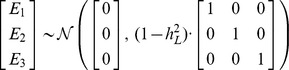
from which it follows that
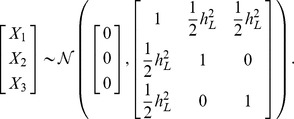



Letting 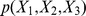 denote the multivariate normal density described above, then the probability, 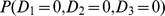, that all three individuals do not have the disease is given by
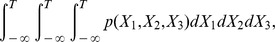
whereas the probability, 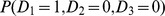, that the index individual develops the disease while his parents do not is
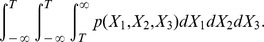
From these two expressions, the risk, 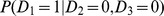, that the index individual will develop the disease given that neither parent develops the disease is
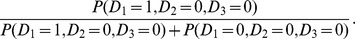



We note that the computational procedures described above, which are identical to the multivariate integration-based method described in Appendix A of the paper from So and colleagues, may be also approximated using results from genetic selection theory [11]. Here, we opted for direct multivariate integration as the computational complexity, though high, was nonetheless tractable for the sizes of pedigrees considered.

Our model is also related to a family-based genetic risk prediction method described by Ruderfer and colleagues, who demonstrated that genetic factors in family members of an index individual can actually be informative of his or her disease risk [12]. As the goal of our study was to compare family history and genetic approaches to risk prediction, we chose specifically to focus on the task of predicting disease risk based on genetic variants in the index individual only and thus excluded the consideration of genetic variants in family members.

#### Assessing discriminative accuracy

For any type of risk prediction model, we may characterize its behavior in several ways:

1. *Sensitivity and specificity.* For a binary classifier, sensitivity and specificity describe the probabilities that a true case (i.e., an index individual with the disease) or a true control (i.e., an index individual without the disease), respectively, will be correctly labeled by the classifier. A risk prediction model corresponds to a family of binary classifiers, parameterized by a threshold 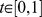: the binary classifier for a given threshold 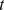 predicts an index individual to be a case if 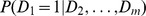 is at least 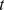 or a control otherwise. For a risk prediction model, sensitivity and specificity must always be considered together as it is otherwise trivial to obtain a classifier with high sensitivity but low specificity (pick a low threshold such that all individuals are labeled cases) or high specificity but low sensitivity (pick a high threshold that such that all individuals are labeled controls).
2. *ROC curve and AUC.* The receiver operating characteristic (ROC) curve for a risk prediction model depicts the trade-off between sensitivity and specificity of the classifiers derived from a risk prediction model at varying thresholds 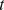. By convention, the points of a ROC curve are specified as 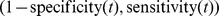 for varying 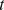. The area under the ROC curve (AUC) is a commonly used summary measure of discriminative accuracy that avoids the need for choosing a single specific threshold 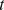. The AUC ranges from 0.5 (random guessing) to 1 (perfect discrimination) and can be interpreted as the probability that a randomly chosen case from the population will have a higher predicted risk than a randomly chosen control.
3. *Positive and negative predictive value.* For a binary classifier, positive predictive value (PPV) and negative predictive value (NPV) refer to the probabilities that a predicted case or a predicted control are actually a true case or a true control, respectively. Like sensitivity and specificity, the PPV and NPV of a risk prediction model vary depending on the specific threshold 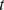 used. Unlike sensitivity and specificity, however, the measured PPV and NPV of a given classifier depend strongly on the pre-test risk 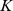 of the condition in the target population.
4. *Likelihood ratio.* Unlike the previous measures which focus on the accuracy of a binary classification algorithm at the population level, likelihood ratios (LRs) quantify the information conveyed by a risk assessment at the individual level. As described in the text, an LR is defined as the ratio of post-test odds to pre-test odds of disease, and the distribution of LRs observed in a population determines the type and frequency of test outcomes one should expect from a risk prediction algorithm.

To analyze the predictive accuracy of family history-based approaches to risk prediction, we describe a direct computational procedure based on examining the expected performance of a “complete” family history-based classifier—i.e., one with full access to the true probability of disease for an index individual for each possible family history pattern. Our procedure works by first enumerating all 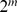 possible patterns of disease occurrence in a family of 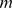 individuals. We explicitly compute the full joint distribution 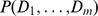 over disease statuses of the index individual and his relatives by evaluating the resulting multivariate Gaussian integrals numerically [41]. This step is generally extremely computationally expensive as numerical integration techniques can be slow and the number of family history patterns that must be considered is exponential in 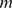; however, for sufficiently small families, this calculation can be performed on a standard desktop computer.

Given the joint distribution over disease statuses, a complete family history-based risk prediction model predicts the disease risk for an individual with family history pattern 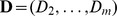 to be exactly 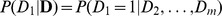. Let 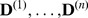 be an enumeration of the 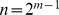 possible family history patterns. To analyze the accuracy of such a model according to the metrics outlined above, observe that for any given 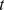, the probability of a true positive (TP), false positive (FP), true negative (TN), or false negative (FN) for a classifier based on threshold 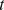 is
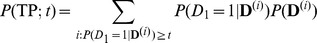


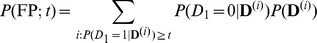


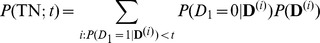


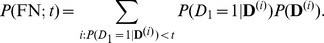
It follows that
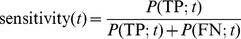


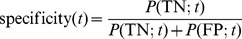


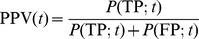


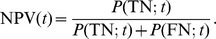



**Figure 5:**
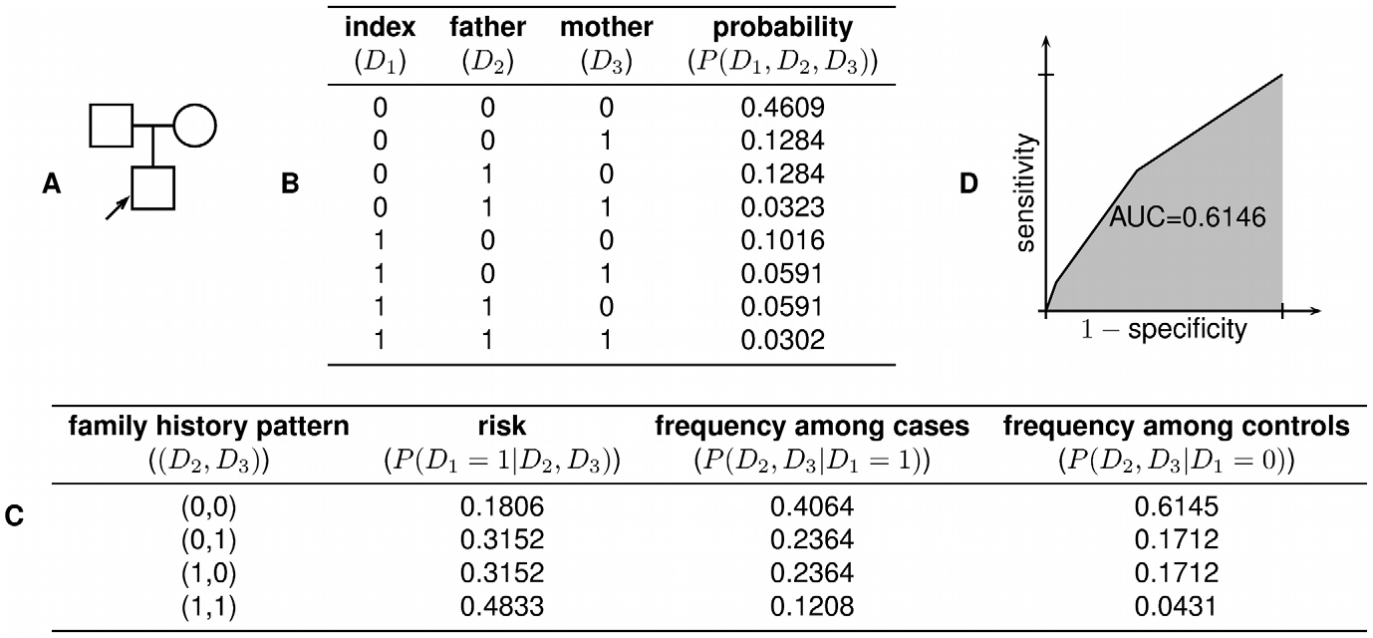
Worked example. (A) In the family structure shown, the shaded box represents the index individual, whose risk of developing the disease we wish to predict. (B) There are 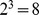 possible combinations of disease status for the individuals in the family. Using the liability threshold model, we compute the probability of each combination; in this example, we assume 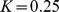 and 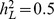. (C) From the joint distribution, we can then compute the disease risk of the index individual for any given family history pattern, as well as the likelihood of particular family history patterns among cases and controls. (D) These quantities then allow us to construct the receiver operating characteristic (ROC) curve for a complete family history-based classifier, from which sensitivity, specificity, PPV, NPV, and AUC can be computed.

To compute the above quantities efficiently, assume without loss of generality that 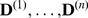 are sorted in order of non-increasing disease risk (i.e., 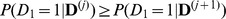 for 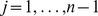). Then, the vertices of the ROC curve are given explicitly by:
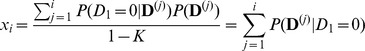


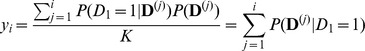
for 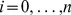. Given any point on the ROC curve corresponding to a 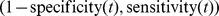 pair, we can easily determine 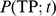 and 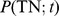 based on the fact that 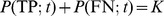 and 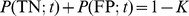. From this, the computation of 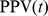 and 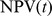 is then straightforward. Finally, the likelihood ratio for any given value of risk 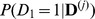 is given by 
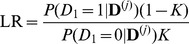
. The key parts of this procedure are illustrated for a small example family in Figure 5.

As a minor technical point, the threshold-based classifiers described above are actually only able to achieve a finite number of combinations of sensitivity and specificity, as given by the vertices of the ROC curve. Nonetheless, using appropriately constructed randomized strategies, the sensitivity and specificity pair corresponding to any convex combination of vertices of the ROC curve can also be realized; the graphs shown in this paper rely on this extension.

#### Complete versus restricted models of family history

Underlying our procedure for estimating the accuracy of a complete family history-based risk prediction model is the premise that the model makes full use of the exact pattern of disease occurrence in the relatives of the index individual. This represents a perfect knowledge scenario in which a genetic counselor has access to a patient's complete family history and has the ability to recognize subtle differences in risk that result from minor differences in patterns of disease occurrence. In clinical practice, however, obtaining a fully accurate family history can be extremely challenging, and making exact inferences based on the information collected can also be very difficult. For example, a genetic counselor may have incomplete or incorrect information regarding a patient's family history as a consequence of misinformation from relatives, errors in patient recollection of his or her family's medical information, or hesitance to share this information openly.

These sources of potential error motivate the use of “restricted” models in which the full family history pattern for an index individual is condensed into a single summary statistic. For example, the Gail model for estimating breast cancer risk considers only the thresholded number of first-degree relatives of the index individual who have the disease (either 0, 1, or 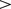
1) [15]. This dramatically reduced model more closely reflects the granularity of information used in a standard clinical setting when a detailed family history is not available.

Formally, we can represent a restricted model by defining a function 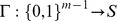, mapping from complete family histories to summary statistics; in the case of the Gail model, for instance, the set 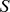 contains exactly three possible outcomes, corresponding to the thresholded number of affected first-degree relatives of the index individual. A restricted family history-based risk prediction model would then estimate that the probability of disease, 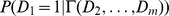, for an index individual with family history pattern 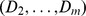 is
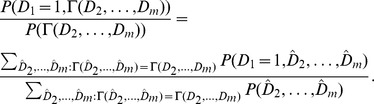
The remainder of the procedures for estimating accuracy are identical to what was described previously with the sole modification that rather than enumerating 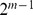 distinct family history patterns for the index individual, we instead enumerate equivalence classes of family history patterns (i.e., distinct values of 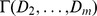).

#### Accounting for sex dependence

The liability threshold model presented here thus far applies generally for diseases where the lifetime risk of the disease does not differ between the sexes. In some scenarios, however, such an assumption may not be realistic. First, a disease may not be applicable for one of the two sexes; for example, women do not develop prostate cancer, nor are men at risk for ovarian cancer. Second, sex differences may inherently reflect the involvement of different genetic and environmental causal factors altogether. Third, heterogeneity in disease frequency between men and women may simply be due to differences in baseline susceptibility, even when the majority of genetic and environmental risk factors are shared between the sexes.

In the first scenario, an appropriate adjustment to our family history-based risk prediction model would be to treat the phenotypic status for individuals of the sex for whom the disease is not relevant as having an unobserved phenotype. As a consequence of the marginalization properties of multivariate Gaussians, such a treatment is mathematically equivalent to removing these individuals from the model altogether. In the second scenario, differences in the underlying disease etiology would essentially require analyzing the two versions of the disease separately. This would involve running male-only and female-only analyses using sex-specific heritability estimates, as described in the first scenario above. The third scenario may be handled by a modification of the liability threshold model to include explicit modeling of sex-specific effects; we do not pursue this direction here but include an explicit derivation (which may be extended to arbitrary discrete covariates) in Text S1.

For the experiments in this paper, we used sex-averaged disease frequencies for most diseases for simplicity. In the case of breast, ovarian, and prostate cancer, we used sex-specific disease frequencies (in particular, ignoring male breast cancer cases) and used the first approach described above. When the sex of the individual shown in the test pedigree was not appropriate for disease being tested, we flipped the sexes of all individuals in the pedigree.

### SNP–based risk assessment models

The estimation of predictive performance for models involving genetic factors has been discussed in detail previously [16], [17]; here, we provide a brief review of the relevant theory.

In the scenario of genetic risk prediction, we assume that known genetic factors account for a fraction 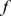 of the total variance of the additive genetic component 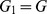 for the index individual; here, we have dropped the subscript “1” as the genetic risk prediction models discussed here do not make use of family information. We model the condition of incomplete genetic information by assuming that 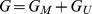, where 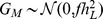 and 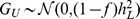 correspond to the measurable and unmeasurable components of additive genetic liability, respectively. Genetic risk prediction, then, is the task of estimating the probability that the index individual will develop disease, given the measurable component of his or her additive genetic liability, i.e., 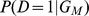. Here, our genetic risk estimates rely only on measured genetic information from the index individual. A related model for family-based genetic risk prediction was previously proposed that also takes into account information from the genetic data for relatives [12].

#### Assessing discriminative accuracy

For the accuracy metrics considered in this paper, computing 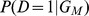 explicitly is unnecessary if the goal is simply to estimate the accuracy of the model. Since 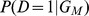 is a strictly increasing monotonic function of 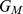, then for any two individuals, the relative ordering of their disease risks is identical to the relative ordering of the measurable components of their respective genetic liabilities.

To apply this fact, suppose that 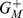 and 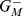 denote the measurable genetic components for a random case and control from the population; that is, suppose that 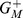 and 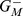 are sampled from 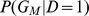 and 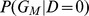, respectively. Standard results from genetic selection theory can be used to prove that
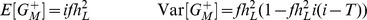


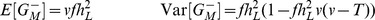
where 
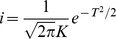
 and 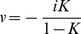. Assuming 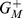 and 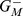 to each be normally distributed [16], [17], then the AUC is simply the integral of the tail distribution of a difference between two normal variables, and thus can be written explicitly as
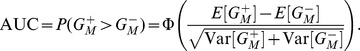
Furthermore, the points on the ROC curve are given by the parametric curve, 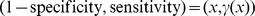 for 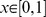, where
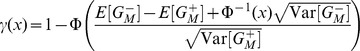
from which other measures of accuracy, such as PPV and NPV, can be derived.

#### Estimating proportion of heritability explained

To estimate the proportion 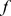 of additive genetic liability accounted for by known SNP factors, we started from a list of curated genome-wide associations compiled by the National Human Genome Research Institute (NHGRI) [1], retrieved on January 18, 2012. From this list, we excluded associations derived in non-European populations, with 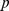-values exceeding the genome-wide significance threshold of 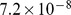 estimated for European studies [42], or referring to SNPs not present in the August 2010 release of the 1000 Genomes dataset [43]. The remaining associations were ordered based on the effective sample size for the study in which the association was reported (harmonic mean of the total numbers of cases and controls), with ties resolved by the strength of evidence for the reported association (in terms of 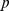-value). Then, for each phenotype separately, a greedy algorithm was used to select a set of associations such that the 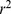 between any two SNPs (as measured on a European subset of 283 individuals from the 1000 Genomes dataset) was at most 0.005, so as to ensure approximate statistical independence of all risk markers.

We then calculated 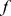 using the technique recently described by So and colleagues [5], which is essentially equivalent to an earlier approach described by Risch [44]. To prevent inflation of variance estimates arising from the “winner's curse” (a systematic inflation of effect sizes from GWAS due to selective reporting of significant associations) [45], we used a bias-correction procedure to adjust the magnitude of reported odds ratios based on the strength of the original association and the threshold applied for significance (assumed to be 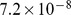, as above) [46].

We also used population allele frequencies based on the European subset of the 1000 Genomes dataset, so as to obtain representative estimates of the proportion of heritability explained in an unselected population; to obtain these population frequencies, we determined allele stranding for all associations in all cases where this could be done unambiguously either on the basis of the stated risk allele in the NHGRI list, or by matching study allele frequencies to frequencies in the 1000 Genomes dataset (provided that the minor allele frequency in each set was at most 0.45); we omitted associations for which neither of the above criteria were met, or where the strand information implied by the two approaches were in conflict. A complete list of the associations used in our models is presented in Table S5.

We note that our estimates of heritability explained are in many instances lower than estimates that have been given elsewhere (see Table 3). These differences may be partially explained by incompleteness or errors in the NHGRI list of associations (due to omitted studies, missing or incorrect annotations, or the fact that not all known verified SNP associations have been discovered through GWAS), omission of rare variants not available in the 1000 Genomes data, or our use of bias-correction and population allele frequencies to provide conservative estimates of SNP-based model accuracies (see Table 3 for a comparison of estimates when omitting one or more of the above corrections).

**Table 3:** Estimated proportion of heritability explained by SNPs for various diseases.

Disease	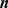	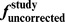	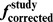	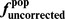	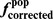	Other estimates
Age-related macular degeneration	9	26.7%	26.2%	26.3%	25.9%	-
Alzheimer disease	10	12.2%	11.2%	10.9%	10.0%	23.22% [5]
Atrial fibrillation	3	5.5%	5.5%	6.3%	6.1%	-
Bipolar disorder	5	9.5%	1.0%	9.5%	1.1%	2.77% [5]
Bladder cancer	8	18.0%	15.9%	18.3%	16.2%	-
Breast cancer	14	17.2%	10.3%	18.2%	10.5%	12.52% [5]
Celiac disease	21	21.8%	21.2%	13.2%	12.6%	40% [47]
Colorectal cancer	11	11.2%	7.9%	12.3%	8.6%	-
Coronary artery disease	24	10.1%	6.8%	10.3%	6.9%	25.15% [5]
Crohn disease	60	15.5%	13.5%	15.5%	13.5%	23.2% [48], 13.43% [5]
Lung cancer	2	0.0%	0.0%	4.7%	2.3%	-
Melanoma	11	48.0%	41.3%	24.4%	19.7%	18.9% [49]
Multiple sclerosis	37	4.1%	2.4%	9.7%	6.1%	17% [50]
Ovarian cancer	3	1.2%	1.2%	2.2%	2.0%	-
Pancreatic cancer	3	2.7%	1.7%	2.8%	1.7%	-
Parkinson disease	10	7.9%	5.8%	8.1%	6.0%	5–7% [51]
Prostate cancer	16	13.0%	10.7%	14.4%	11.9%	31.16% [5]
Schizophrenia	8	2.1%	0.4%	2.0%	0.4%	0.39% [5]
Stroke	1	2.0%	1.5%	2.5%	1.9%	0% [52]
Thyroid cancer	2	4.8%	4.3%	4.9%	4.3%	-
Type 1 diabetes	26	6.0%	4.8%	5.8%	4.5%	13.63% [5]
Type 2 diabetes	21	2.0%	0.7%	17.2%	13.0%	27.93% [5]
Ulcerative colitis	37	9.7%	9.0%	9.9%	9.2%	16% [53]

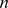
 indicates the number of SNP markers used in the model being evaluated. All other values indicate the estimated proportion of variance in additive genetic liability accounted for by known SNP associations. The columns labeled with 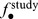 rely on the risk allele frequencies in the control populations, as provided in the NHGRI association list, whereas the columns labeled with 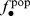 rely on population allele frequencies in the 1000 Genomes dataset. The columns labeled with 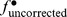 use the raw odds ratios provided in the NHGRI association list, whereas the columns labeled with 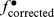 use odds ratios that have been adjusted for winner's curse bias. The final column provides references for other estimates of 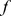 from the literature.

### Combining family history and SNP–based models

Consider a further extension of the liability threshold model for family history in which the genetic liability for each individual is decomposed as 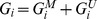. Here, 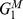 represents the measured genetic liability based on SNPs found in the index individual, and 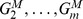 represent the corresponding components of genetic liability in other individuals from the family. As before, 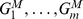 have a joint multivariate normal distribution with zero mean and covariance matrix 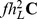, and 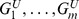 have a joint multivariate normal distribution with zero mean and covariance matrix 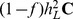.

Decompose the matrix 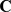 as
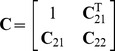
where 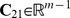, and 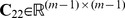. The conditional distribution of 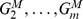 given the measured genetic liability for the index individual follows from standard formulas for conditional Gaussians:
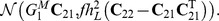
Based on the above result and the fact that 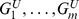 and 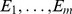 are independent of 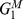, it can be shown that the conditional distribution of total liabilities 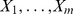 given 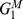 is
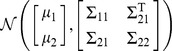
where
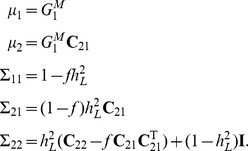
As described previously, we can use multivariate numerical integration procedures to evaluate the conditional distribution 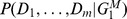 over diseases statuses corresponding to the above joint distribution over total liabilities; this conditional distribution, in turn, may be used to evaluate the genotype-specific risks 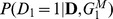 for each possible family history pattern, and the genotype-specific frequencies 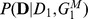 of each pattern among cases and controls.

To estimate the AUC for a combined family history and SNP-based model, we use a discrete approximation for the distribution of 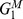; specifically, we assume that values of 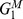 are drawn uniformly at random from the finite set 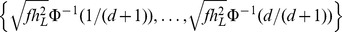. The accuracy of this approximation improves as 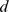 increases; in practice, we use 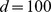. Enumerating the possible values of 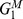 in this way allows us to explicitly compute the full joint distribution 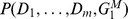.

Let 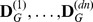 be an enumeration of the 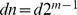 possible family history patterns conditioned on measured genotype of the index individual; here, each 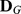 can be thought of as a pair consisting of both the family history pattern 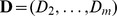 for the index individual and his measured genetic liability 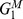. The formulas provided previously for identifying points on the ROC curve generalize immediately. We point out that this treatment does not assume independence of the risks conferred by family history and measured genetic contributions, and may be easily generalized to situations where genetic measurements in a subset of relatives are also known (see also [12]).

### Accounting for shared environment

Within a family, a simple way of upper-bounding the impact of shared environment would be to assume a common component of liability due to shared environment among all family members. This assumption may not be particularly realistic, especially when dealing with large pedigrees such as shown in Figure 1, given that it attributes the same amount of shared environmental variance between all pairs of individuals in the pedigree (i.e., siblings share as much as environment as more distant relatives). In the absence of more realistic estimates of environmental sharing, however, this assumption provides a tractable hypothesis that allows computation of accuracy upper bounds. If the liabilities for individuals in a family are decomposed as 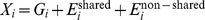, and the proportion of variance in total liability due to shared environmental factors is 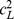, then 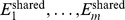 have a degenerate joint multivariate normal distribution with zero mean and singular covariance matrix 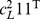. It follows that 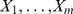 have a joint multivariate normal distribution with zero mean and covariance matrix 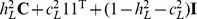; integrating this joint multivariate normal distribution, as before, allows us to compute estimates of accuracy for family history-based models that take into account common shared environment. An analogous procedure may be applied for combined family history and SNP-based models.

## Supporting Information

Figure S1 Additional test pedigrees. The pedigrees shown correspond to (A) a trio, (B) a nuclear family with multiple children, and (C) an extended family with four grandparents. In each pedigree, an arrow designates one particular individual as the “index individual” (or consultand) whose disease risk we wish to predict.
(EPS)

Figure S2 Additional AUC plots. Plots of AUC for pedigrees (A), (B), and (C) from Figure S1.
(EPS)

Figure S3 Additional LR distribution plots. Plots of likelihood ratio distribution for pedigrees (A), (B), and (C) from Figure S1.
(EPS)

Figure S4Receiver operating characteristic (ROC) plots. ROC plots pedigrees (A), (B), and (C) from Figure S1 and the test pedigree (D) from Figure 1. Within each plot, the subplots show the relationship between 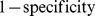 (on the horizontal axis) and sensitivity (on the vertical axis) at varying risk thresholds. The curves in each subplot represent different prediction models, including a complete family history model that accounts for the disease status of every individual in the pedigree (solid red line), a restricted family history model that only considers the number (0, 1, or 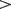
1) of affected first-degree relatives of the index individual (dashed red line), and genetic factors accounting for either 10% (dotted blue line), 30% (dashed blue line), or 100% (solid blue line) of the heritability of the disease.(EPS)

Figure S5Positive predictive value (PPV) plots. Plots of PPV for pedigrees (A), (B), and (C) from Figure S1 and the test pedigree (D) from Figure 1. Within each plot, the subplots show the relationship between sensitivity (on the horizontal axis) and PPV (on the vertical axis) at the risk threshold needed to achieve the specified sensitivity. The curves in each subplot represent different prediction models, including a complete family history model that accounts for the disease status of every individual in the pedigree (solid red line), a restricted family history model that only considers the number (0, 1, or 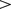
1) of affected first-degree relatives of the index individual (dashed red line), and genetic factors accounting for either 10% (dotted blue line), 30% (dashed blue line), or 100% (solid blue line) of the heritability of the disease.(EPS)

Figure S6Negative predictive value (NPV) plots. Plots of NPV for pedigrees (A), (B), and (C) from Figure S1 and the test pedigree (D) from Figure 1. Within each plot, the subplots show the relationship between sensitivity (on the horizontal axis) and NPV (on the vertical axis) at the risk threshold needed to achieve the specified sensitivity. The curves in each plot represent different prediction models, including a complete family history model that accounts for the disease status of every individual in the pedigree (solid red line), a restricted family history model that only considers the number (0, 1, or 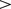
1) of affected first-degree relatives of the index individual (dashed red line), and genetic factors accounting for either 10% (dotted blue line), 30% (dashed blue line), or 100% (solid blue line) of the heritability of the disease.(EPS)

Table S1Performance summary for family history. 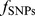 provides the proportion of heritability explained by known SNP associations. The last eight columns indicate the 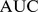 achieved by complete and restricted family history models using either the test pedigree in Figure 1 or the additional pedigrees in parts (A), (B), and (C) of Figure S1. Bold entries indicate situations where the SNP-based model (see accuracies in Table 2) based on currently known associations outperforms family history. Note that the performance of models shown here reflects only currently known genetic factors for European populations and will change as more associations are discovered.(PDF)

Table S2Performance summary for family history, including shared environment. 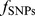 provides the proportion of heritability explained by known SNP associations. The last eight columns indicate the 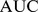 achieved by complete and restricted family history models, assuming 10% of the variance in liability is due to shared environment across all individuals in the family, using either the test pedigree in Figure 1 or the additional pedigrees in parts (A), (B), and (C) of Figure S1. Bold entries indicate situations where the SNP-based model (see accuracies in Table 2) based on currently known associations outperforms family history. Note that the performance of models shown here reflects only currently known genetic factors for European populations and will change as more associations are discovered.(PDF)

Table S3Performance summary for combining family history and SNPs. 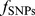 provides the proportion of heritability explained by known SNP associations. The last eight columns indicate the 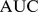 achieved when combining these SNP associations with complete and restricted family history models using either the test pedigree in Figure 1 or the additional pedigrees in parts (A), (B), and (C) of Figure S1. Note that the performance of models shown here reflects only currently known genetic factors for European populations and will change as more associations are discovered.(PDF)

Table S4Performance summary for combining family history and SNPs, including shared environment. 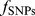 provides the proportion of heritability explained by known SNP associations. The last eight columns indicate the 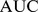 achieved when combining these SNP associations with complete and restricted family history models, assuming 10% of the variance in liability is due to shared environment across all individuals in the family, using either the test pedigree in Figure 1 or the additional pedigrees in parts (A), (B), and (C) of Figure S1. Note that the performance of models shown here reflects only currently known genetic factors for European populations and will change as more associations are discovered.(PDF)

Table S5Associations used to assess SNP-based risk models. Alleles are indicated as reference allele/variant allele. 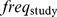 and 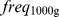 provide the variant allele frequencies according to the NHGRI list and based on the 1000 Genomes data, respectively. 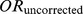 and 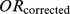 provide the original allelic odds ratio reported on the NHGRI list and its corresponding bias-corrected odds ratio.(PDF)

Text S1 Additional derivations.
(PDF)

Text S2 References for disease frequency and heritability.
(PDF)
