# Supplementary figures and images for: Comparison of Family History and SNPs for Predicting Risk of Complex Disease
Source: PLoS Genet. 2012 Oct 11;8(10):e1002973. doi: 10.1371/journal.pgen.1002973 (PMC3469463; doi:10.1371/journal.pgen.1002973)

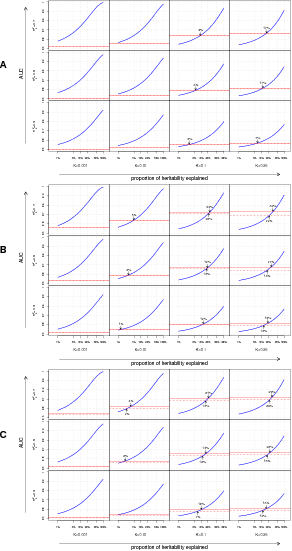

Supplement: Figure S2 — Additional AUC plots. Plots of AUC for pedigrees (A), (B), and (C) from Figure S1. (EPS) [file pgen.1002973.s002.tif]
