# Supplementary material for: Comparison of Family History and SNPs for Predicting Risk of Complex Disease
Source: PLoS Genet. 2012 Oct 11;8(10):e1002973. doi: 10.1371/journal.pgen.1002973 (PMC3469463; doi:10.1371/journal.pgen.1002973)
Supplement: Table S1 — Performance summary for family history. provides the proportion of heritability explained by known SNP associations. The last eight columns indicate the achieved by complete and restricted family history models using either the test pedigree in Figure 1 or the additional pedigrees in parts (A), (B), and (C) of Figure S1. Bold entries indicate situations where the SNP-based model (see accuracies in Table 2) based on currently known associations outperforms family history. Note that the performance of models shown here reflects only currently known genetic factors for European populations and will change as more associations are discovered. (PDF) [file pgen.1002973.s007.pdf]

Table S1: **Performance summary for family history.**

				1		S1A		S1B		S1C	
Disease	K	h_L^2	f_{SNPs}	complete	restricted	complete	restricted	complete	restricted	complete	restricted
Coronary artery disease	0.402	0.49	6.9%	0.665	0.649	0.618	0.618	0.673	0.672	0.662	0.649
Type 2 diabetes	0.339	0.30	13.0%	0.610	0.596	**0.571**	**0.571**	0.616	0.616	0.606	0.596
Atrial fibrillation	0.245	0.62	6.1%	0.701	0.681	0.644	0.644	0.709	0.708	0.697	0.681
Stroke	0.190	0.17	1.9%	0.564	0.552	0.535	0.535	0.568	0.568	0.561	0.552
Prostate cancer	0.165	0.42	11.9%	**0.606**	**0.583**	**0.552**	**0.552**	**0.604**	**0.604**	**0.601**	**0.583**
Alzheimer disease	0.132	0.79	10.0%	0.743	0.715	0.670	0.670	0.750	0.748	0.737	0.715
Breast cancer	0.123	0.25	10.5%	**0.560**	**0.544**	**0.526**	**0.526**	**0.558**	**0.558**	**0.557**	**0.544**
Lung cancer	0.069	0.08	2.3%	0.527	**0.519**	**0.511**	**0.511**	0.528	0.528	**0.524**	**0.519**
Bipolar disorder	0.051	0.60	1.1%	0.675	0.637	0.592	0.592	0.679	0.679	0.664	0.637
Colorectal cancer	0.051	0.13	8.6%	**0.538**	**0.526**	**0.515**	**0.515**	**0.539**	**0.539**	**0.534**	**0.526**
Age-related macular degeneration	0.047	0.71	25.9%	**0.700**	**0.661**	**0.612**	**0.612**	**0.705**	**0.705**	**0.689**	**0.661**
Bladder cancer	0.024	0.08	16.2%	**0.517**	**0.511**	**0.506**	**0.506**	**0.517**	**0.517**	**0.515**	**0.511**
Multiple sclerosis	0.020	0.51	6.1%	**0.615**	**0.582**	**0.549**	**0.549**	**0.618**	**0.618**	**0.604**	**0.582**
Melanoma	0.020	0.21	19.7%	**0.544**	**0.528**	**0.515**	**0.515**	**0.544**	**0.544**	**0.538**	**0.528**
Type 1 diabetes	0.018	0.87	4.5%	0.700	0.660	**0.610**	**0.610**	0.710	0.709	0.688	0.660
Parkinson disease	0.016	0.27	6.0%	**0.553**	**0.534**	**0.518**	**0.518**	**0.553**	**0.553**	**0.546**	**0.534**
Pancreatic cancer	0.015	0.36	1.7%	0.569	**0.545**	**0.525**	**0.525**	0.570	0.570	0.560	**0.545**
Ovarian cancer	0.014	0.22	2.0%	**0.520**	**0.513**	**0.507**	**0.507**	**0.519**	**0.519**	**0.518**	**0.513**
Thyroid cancer	0.010	0.53	4.3%	**0.591**	**0.563**	**0.536**	**0.536**	**0.595**	**0.595**	**0.580**	**0.563**
Ulcerative colitis	0.009	0.53	9.2%	**0.588**	**0.561**	**0.535**	**0.535**	**0.593**	**0.593**	**0.578**	**0.561**
Schizophrenia	0.007	0.66	0.4%	0.607	0.578	0.546	0.546	0.614	0.614	0.596	0.578
Celiac disease	0.007	0.75	12.6%	**0.624**	**0.594**	**0.557**	**0.557**	**0.635**	**0.634**	**0.613**	**0.594**
Crohn disease	0.005	0.56	13.5%	**0.573**	**0.551**	**0.529**	**0.529**	**0.579**	**0.579**	**0.565**	**0.551**

f_{SNPs} provides the proportion of heritability explained by known SNP associations. The last eight columns indicate the AUC achieved by complete and restricted family history models using either the test pedigree in Figure 1 or the additional pedigrees in parts (A), (B), and (C) of Figure S1. Bold entries indicate situations where the SNP-based model (see accuracies in Table 2) based on currently known associations outperforms family history. Note that the performance of models shown here reflects only currently known genetic factors for European populations and will change as more associations are discovered.
