# Supplementary material for: Comparison of Family History and SNPs for Predicting Risk of Complex Disease
Source: PLoS Genet. 2012 Oct 11;8(10):e1002973. doi: 10.1371/journal.pgen.1002973 (PMC3469463; doi:10.1371/journal.pgen.1002973)
Supplement: Table S2 — Performance summary for family history, including shared environment. provides the proportion of heritability explained by known SNP associations. The last eight columns indicate the achieved by complete and restricted family history models, assuming 10% of the variance in liability is due to shared environment across all individuals in the family, using either the test pedigree in Figure 1 or the additional pedigrees in parts (A), (B), and (C) of Figure S1. Bold entries indicate situations where the SNP-based model (see accuracies in Table 2) based on currently known associations outperforms family history. Note that the performance of models shown here reflects only currently known genetic factors for European populations and will change as more associations are discovered. (PDF) [file pgen.1002973.s008.pdf]

Table S2: **Performance summary for family history, including shared environment.**

				1		S1A		S1B		S1C	
Disease	K	h_L^2	f_{SNPs}	complete	restricted	complete	restricted	complete	restricted	complete	restricted
Coronary artery disease	0.402	0.49	6.9%	0.706	0.684	0.649	0.649	0.709	0.708	0.702	0.684
Type 2 diabetes	0.339	0.30	13.0%	0.663	0.638	0.606	0.606	0.663	0.662	0.657	0.638
Atrial fibrillation	0.245	0.62	6.1%	0.737	0.712	0.672	0.672	0.740	0.739	0.732	0.712
Stroke	0.190	0.17	1.9%	0.632	0.600	0.571	0.571	0.625	0.625	0.623	0.600
Prostate cancer	0.165	0.42	11.9%	0.650	**0.613**	**0.573**	**0.573**	0.639	0.639	0.643	**0.613**
Alzheimer disease	0.132	0.79	10.0%	0.774	0.742	0.698	0.698	0.776	0.775	0.768	0.742
Breast cancer	0.123	0.25	10.5%	0.611	**0.576**	**0.546**	**0.546**	0.597	0.597	0.603	**0.576**
Lung cancer	0.069	0.08	2.3%	0.601	0.563	0.539	0.539	0.587	0.587	0.589	0.563
Bipolar disorder	0.051	0.60	1.1%	0.722	0.674	0.623	0.623	0.718	0.717	0.709	0.674
Colorectal cancer	0.051	0.13	8.6%	0.608	0.567	**0.540**	**0.540**	0.594	0.594	0.594	0.567
Age-related macular degeneration	0.047	0.71	25.9%	**0.743**	**0.695**	**0.642**	**0.642**	**0.740**	**0.740**	**0.731**	**0.695**
Bladder cancer	0.024	0.08	16.2%	0.580	**0.542**	**0.524**	**0.524**	**0.564**	**0.564**	**0.566**	**0.542**
Multiple sclerosis	0.020	0.51	6.1%	0.671	**0.619**	**0.575**	**0.575**	0.663	0.663	0.654	**0.619**
Melanoma	0.020	0.21	19.7%	**0.603**	**0.561**	**0.535**	**0.535**	**0.590**	**0.590**	**0.588**	**0.561**
Type 1 diabetes	0.018	0.87	4.5%	0.746	0.696	0.642	0.642	0.746	0.746	0.732	0.696
Parkinson disease	0.016	0.27	6.0%	0.610	**0.566**	**0.538**	**0.538**	0.599	0.599	0.595	**0.567**
Pancreatic cancer	0.015	0.36	1.7%	0.625	0.579	**0.546**	**0.546**	0.615	0.615	0.609	0.579
Ovarian cancer	0.014	0.22	2.0%	0.553	**0.530**	**0.516**	**0.516**	**0.542**	**0.542**	**0.546**	**0.530**
Thyroid cancer	0.010	0.53	4.3%	0.645	**0.598**	**0.559**	**0.559**	0.640	0.640	0.629	**0.598**
Ulcerative colitis	0.009	0.53	9.2%	**0.642**	**0.596**	**0.558**	**0.558**	**0.637**	**0.637**	**0.626**	**0.596**
Schizophrenia	0.007	0.66	0.4%	0.660	0.613	0.571	0.571	0.658	0.658	0.644	0.613
Celiac disease	0.007	0.75	12.6%	**0.677**	**0.630**	**0.584**	**0.584**	**0.678**	**0.678**	**0.661**	**0.630**
Crohn disease	0.005	0.56	13.5%	**0.624**	**0.583**	**0.549**	**0.549**	**0.621**	**0.621**	**0.609**	**0.583**

f_{SNPs} provides the proportion of heritability explained by known SNP associations. The last eight columns indicate the AUC achieved by complete and restricted family history models, assuming 10% of the variance in liability is due to shared environment across all individuals in the family, using either the test pedigree in Figure 1 or the additional pedigrees in parts (A), (B), and (C) of Figure S1. Bold entries indicate situations where the SNP-based model (see accuracies in Table 2) based on currently known associations outperforms family history. Note that the performance of models shown here reflects only currently known genetic factors for European populations and will change as more associations are discovered.
