# Supplementary material for: Comparison of Family History and SNPs for Predicting Risk of Complex Disease
Source: PLoS Genet. 2012 Oct 11;8(10):e1002973. doi: 10.1371/journal.pgen.1002973 (PMC3469463; doi:10.1371/journal.pgen.1002973)
Supplement: Table S3 — Performance summary for combining family history and SNPs. provides the proportion of heritability explained by known SNP associations. The last eight columns indicate the achieved when combining these SNP associations with complete and restricted family history models using either the test pedigree in Figure 1 or the additional pedigrees in parts (A), (B), and (C) of Figure S1. Note that the performance of models shown here reflects only currently known genetic factors for European populations and will change as more associations are discovered. (PDF) [file pgen.1002973.s009.pdf]

Table S3: **Performance summary for combining family history and SNPs.**

				1		S1A		S1B		S1C	
Disease	K	h^2_L	f_{SNPs}	complete	restricted	complete	restricted	complete	restricted	complete	restricted
Coronary artery disease	0.402	0.49	6.9%	0.675	0.666	0.645	0.645	0.683	0.683	0.673	0.666
Type 2 diabetes	0.339	0.30	13.0%	0.631	0.624	0.612	0.612	0.637	0.637	0.629	0.624
Atrial fibrillation	0.245	0.62	6.1%	0.712	0.701	0.677	0.677	0.721	0.721	0.709	0.701
Stroke	0.190	0.17	1.9%	0.569	0.561	0.549	0.549	0.574	0.574	0.566	0.561
Prostate cancer	0.165	0.42	11.9%	0.648	0.641	0.628	0.628	0.651	0.651	0.646	0.641
Alzheimer disease	0.132	0.79	10.0%	0.767	0.756	0.732	0.732	0.777	0.777	0.764	0.756
Breast cancer	0.123	0.25	10.5%	0.602	0.598	0.592	0.592	0.604	0.604	0.601	0.598
Lung cancer	0.069	0.08	2.3%	0.537	0.534	0.530	0.530	0.539	0.539	0.536	0.534
Bipolar disorder	0.051	0.60	1.1%	0.687	0.661	0.624	0.624	0.696	0.696	0.680	0.661
Colorectal cancer	0.051	0.13	8.6%	0.573	0.570	0.566	0.566	0.575	0.575	0.572	0.570
Age-related macular degeneration	0.047	0.71	25.9%	0.798	0.792	0.778	0.778	0.805	0.805	0.796	0.792
Bladder cancer	0.024	0.08	16.2%	0.577	0.576	0.575	0.575	0.577	0.577	0.577	0.576
Multiple sclerosis	0.020	0.51	6.1%	0.678	0.665	0.646	0.646	0.687	0.687	0.673	0.665
Melanoma	0.020	0.21	19.7%	0.644	0.642	0.639	0.639	0.646	0.646	0.643	0.642
Type 1 diabetes	0.018	0.87	4.5%	0.758	0.740	0.706	0.706	0.772	0.772	0.753	0.740
Parkinson disease	0.016	0.27	6.0%	0.612	0.606	0.598	0.598	0.617	0.617	0.610	0.606
Pancreatic cancer	0.015	0.36	1.7%	0.603	0.589	0.574	0.574	0.608	0.608	0.598	0.589
Ovarian cancer	0.014	0.22	2.0%	0.559	0.556	0.551	0.551	0.560	0.560	0.558	0.556
Thyroid cancer	0.010	0.53	4.3%	0.664	0.651	0.634	0.634	0.672	0.672	0.659	0.651
Ulcerative colitis	0.009	0.53	9.2%	0.700	0.691	0.678	0.678	0.707	0.707	0.696	0.691
Schizophrenia	0.007	0.66	0.4%	0.633	0.608	0.580	0.580	0.641	0.641	0.624	0.608
Celiac disease	0.007	0.75	12.6%	0.773	0.765	0.749	0.749	0.783	0.783	0.770	0.765
Crohn disease	0.005	0.56	13.5%	0.735	0.730	0.721	0.721	0.741	0.741	0.733	0.730

f_{SNPs} provides the proportion of heritability explained by known SNP associations. The last eight columns indicate the AUC achieved when combining these SNP associations with complete and restricted family history models using either the test pedigree in Figure 1 or the additional pedigrees in parts (A), (B), and (C) of Figure S1. Note that the performance of models shown here reflects only currently known genetic factors for European populations and will change as more associations are discovered.
