# Supplementary material for: Comparison of Family History and SNPs for Predicting Risk of Complex Disease
Source: PLoS Genet. 2012 Oct 11;8(10):e1002973. doi: 10.1371/journal.pgen.1002973 (PMC3469463; doi:10.1371/journal.pgen.1002973)
Supplement: Table S4 — Performance summary for combining family history and SNPs, including shared environment. provides the proportion of heritability explained by known SNP associations. The last eight columns indicate the achieved when combining these SNP associations with complete and restricted family history models, assuming 10% of the variance in liability is due to shared environment across all individuals in the family, using either the test pedigree in Figure 1 or the additional pedigrees in parts (A), (B), and (C) of Figure S1. Note that the performance of models shown here reflects only currently known genetic factors for European populations and will change as more associations are discovered. (PDF) [file pgen.1002973.s010.pdf]

Table S4: **Performance summary for combining family history and SNPs, including shared environment.**

				1		S1A		S1B		S1C	
Disease	K	h_L^2	f_{SNPs}	complete	restricted	complete	restricted	complete	restricted	complete	restricted
Coronary artery disease	0.402	0.49	6.9%	0.729	0.713	0.688	0.688	0.733	0.732	0.725	0.713
Type 2 diabetes	0.339	0.30	13.0%	0.688	0.670	0.648	0.648	0.688	0.688	0.683	0.670
Atrial fibrillation	0.245	0.62	6.1%	0.763	0.747	0.720	0.720	0.768	0.768	0.760	0.747
Stroke	0.190	0.17	1.9%	0.645	0.617	0.590	0.590	0.640	0.640	0.636	0.617
Prostate cancer	0.165	0.42	11.9%	0.692	0.674	0.650	0.650	0.690	0.690	0.688	0.674
Alzheimer disease	0.132	0.79	10.0%	0.814	0.799	0.774	0.774	0.820	0.819	0.811	0.799
Breast cancer	0.123	0.25	10.5%	0.647	0.628	0.610	0.610	0.642	0.642	0.642	0.628
Lung cancer	0.069	0.08	2.3%	0.614	0.579	0.558	0.558	0.602	0.602	0.603	0.579
Bipolar disorder	0.051	0.60	1.1%	0.747	0.708	0.662	0.662	0.747	0.747	0.737	0.708
Colorectal cancer	0.051	0.13	8.6%	0.635	0.606	0.588	0.588	0.626	0.626	0.625	0.606
Age-related macular degeneration	0.047	0.71	25.9%	0.836	0.822	0.803	0.803	0.840	0.840	0.832	0.822
Bladder cancer	0.024	0.08	16.2%	0.621	0.599	0.588	0.588	0.612	0.612	0.613	0.599
Multiple sclerosis	0.020	0.51	6.1%	0.730	0.700	0.670	0.670	0.731	0.731	0.720	0.700
Melanoma	0.020	0.21	19.7%	0.682	0.663	0.651	0.651	0.677	0.677	0.675	0.663
Type 1 diabetes	0.018	0.87	4.5%	0.808	0.781	0.744	0.744	0.816	0.816	0.801	0.781
Parkinson disease	0.016	0.27	6.0%	0.662	0.634	0.614	0.614	0.657	0.657	0.652	0.634
Pancreatic cancer	0.015	0.36	1.7%	0.658	0.621	0.594	0.594	0.652	0.652	0.645	0.621
Ovarian cancer	0.014	0.22	2.0%	0.589	0.571	0.559	0.559	0.581	0.581	0.584	0.571
Thyroid cancer	0.010	0.53	4.3%	0.712	0.682	0.654	0.654	0.712	0.712	0.701	0.682
Ulcerative colitis	0.009	0.53	9.2%	0.740	0.718	0.695	0.695	0.742	0.742	0.732	0.718
Schizophrenia	0.007	0.66	0.4%	0.687	0.647	0.607	0.607	0.689	0.689	0.673	0.647
Celiac disease	0.007	0.75	12.6%	0.810	0.792	0.769	0.769	0.816	0.816	0.804	0.792
Crohn disease	0.005	0.56	13.5%	0.767	0.750	0.734	0.734	0.769	0.769	0.761	0.750

f_{SNPs} provides the proportion of heritability explained by known SNP associations. The last eight columns indicate the AUC achieved when combining these SNP associations with complete and restricted family history models, assuming 10% of the variance in liability is due to shared environment across all individuals in the family, using either the test pedigree in Figure 1 or the additional pedigrees in parts (A), (B), and (C) of Figure S1. Note that the performance of models shown here reflects only currently known genetic factors for European populations and will change as more associations are discovered.
