# Supplementary material for: Comparison of Family History and SNPs for Predicting Risk of Complex Disease
Source: PLoS Genet. 2012 Oct 11;8(10):e1002973. doi: 10.1371/journal.pgen.1002973 (PMC3469463; doi:10.1371/journal.pgen.1002973)
Supplement: Table S5 — Associations used to assess SNP-based risk models. Alleles are indicated as reference allele/variant allele. and provide the variant allele frequencies according to the NHGRI list and based on the 1000 Genomes data, respectively. and provide the original allelic odds ratio reported on the NHGRI list and its corresponding bias-corrected odds ratio. (PDF) [file pgen.1002973.s011.pdf]

Table S5: **Associations used to assess SNP-based risk models.**

Disease	PubMed ID	SNP	p-value	Alleles	$freq_{\text{study}}$	$freq_{\text{1000g}}$	$OR_{\text{uncorrected}}$	$OR_{\text{corrected}}$
Age-related macular degeneration	21665990	rs10490924	4×10^{-322}	G/T	0.21	0.20	2.94	2.94
	21665990	rs1061170	1×10^{-261}	T/C	0.37	0.38	2.41	2.41
	21665990	rs641153	6×10^{-31}	A/G	0.90	0.92	1.85	1.85
	21665990	rs2230199	5×10^{-29}	G/C	0.19	0.20	1.53	1.53
	21665990	rs9332739	2×10^{-23}	C/G	0.96	0.95	2.17	2.17
	21665990	rs10468017	3×10^{-12}	T/C	0.71	0.73	1.19	1.18
	21665990	rs10033900	4×10^{-10}	C/T	0.46	0.48	1.18	1.15
	21665990	rs3764261	7×10^{-09}	C/A	0.33	0.28	1.15	1.09
	21665990	rs1999930	1×10^{-08}	T/C	0.70	0.70	1.15	1.08
Alzheimer disease	21460840	rs3764650	5×10^{-17}	T/G	0.10	0.11	1.23	1.23
	21460840	rs610932	2×10^{-14}	T/G	0.58	0.51	1.11	1.11
	21460840	rs744373	3×10^{-14}	A/G	0.29	0.30	1.17	1.17
	21460840	rs3818361	4×10^{-14}	G/A	0.19	0.21	1.18	1.18
	21460841	rs561655	7×10^{-11}	G/A	0.66	0.63	1.15	1.13
	21460841	rs11767557	6×10^{-10}	C/T	0.81	0.80	1.11	1.09
	21460841	rs3865444	2×10^{-09}	A/C	0.70	0.69	1.10	1.07
	21460841	rs9349407	9×10^{-09}	G/C	0.27	0.27	1.11	1.06
	20460622	rs2075650	1×10^{-295}	A/G	0.14	0.12	2.53	2.53
	20885792	rs11754661	2×10^{-10}	G/A	0.07	0.06	2.10	1.91
Atrial fibrillation	19597491	rs2200733	1×10^{-14}	C/T	0.12	0.14	1.42	1.42
	19597491	rs7193343	1×10^{-10}	C/T	-	0.17	1.21	1.19
	20173747	rs13376333	2×10^{-21}	C/T	0.30	0.30	1.52	1.52
Bipolar disorder	21926972	rs12576775	4×10^{-08}	A/G	-	0.17	1.14	1.03
	21353194	rs1064395	2×10^{-09}	G/A	0.16	0.16	1.17	1.12
	18711365	rs10994336	9×10^{-09}	C/T	0.05	0.08	1.45	1.24
	17554300	rs420259	6×10^{-08}	G/A	0.72	0.76	2.08	1.19
	17486107	rs1012053	2×10^{-08}	C/A	0.84	0.83	1.59	1.19
Bladder cancer	20972438	rs9642880	2×10^{-18}	G/T	0.45	0.46	1.21	1.21
	20972438	rs798766	4×10^{-13}	C/T	0.19	0.21	1.20	1.19
	20972438	rs1014971	8×10^{-12}	C/T	0.62	0.66	1.18	1.17
	20972438	rs8102137	2×10^{-11}	T/C	0.33	0.28	1.13	1.12
	20972438	rs2294008	4×10^{-11}	C/T	0.46	0.42	1.13	1.12
	20972438	rs1495741	4×10^{-11}	G/A	0.80	0.75	1.15	1.14
	20972438	rs710521	2×10^{-10}	C/T	0.73	0.70	1.18	1.15
	21750109	rs17674580	8×10^{-11}	C/T	0.33	0.33	1.17	1.15
Breast cancer	17529967	rs2981582	2×10^{-76}	G/A	0.38	0.43	1.26	1.26
	17529967	rs3803662	1×10^{-36}	G/A	0.25	0.27	1.20	1.20
	17529967	rs889312	7×10^{-20}	A/C	0.28	0.31	1.13	1.13
	17529967	rs13281615	5×10^{-12}	A/G	0.40	0.45	1.08	1.08
	17529967	rs3817198	3×10^{-09}	T/C	0.30	0.31	1.07	1.05
	20453838	rs614367	3×10^{-15}	C/T	0.15	0.14	1.15	1.15
	20453838	rs10995190	5×10^{-15}	A/G	0.85	0.86	1.16	1.16
	20453838	rs1011970	3×10^{-08}	G/T	0.17	0.19	1.09	1.02
	21263130	rs4415084	8×10^{-11}	C/T	0.42	0.41	1.17	1.15
	21263130	rs865686	2×10^{-10}	G/T	0.61	0.65	1.12	1.10
	21263130	rs4973768	2×10^{-08}	T/C	0.49	0.49	1.14	1.05
	19330030	rs11249433	7×10^{-10}	A/G	0.39	0.43	1.16	1.13
	20852631	rs8170	2×10^{-09}	G/A	0.17	0.17	1.26	1.19
	18326623	rs2180341	3×10^{-08}	A/G	0.21	0.27	1.41	1.10
Celiac disease	20190752	rs2187668	1×10^{-50}	C/T	0.26	0.09	6.23	6.23
	20190752	rs1464510	3×10^{-40}	C/A	0.49	0.48	1.29	1.29
	20190752	rs17810546	4×10^{-28}	A/G	0.13	0.09	1.36	1.36
	20190752	rs2816316	2×10^{-17}	C/A	0.84	0.80	1.25	1.25
	20190752	rs13098911	3×10^{-17}	C/T	0.10	0.10	1.30	1.30
	20190752	rs11221332	5×10^{-16}	C/T	0.24	0.24	1.21	1.21
	20190752	rs917997	1×10^{-15}	C/T	0.24	0.23	1.19	1.19
	20190752	rs1738074	3×10^{-15}	C/T	0.43	0.46	1.16	1.16
	20190752	rs802734	3×10^{-14}	A/G	0.31	0.28	1.17	1.17
	20190752	rs13003464	4×10^{-13}	A/G	0.40	0.35	1.15	1.15
	20190752	rs10903122	2×10^{-10}	A/G	0.52	0.55	1.12	1.10
	20190752	rs1893217	3×10^{-10}	A/G	0.17	0.14	1.17	1.14
	20190752	rs10806425	4×10^{-10}	C/A	0.40	0.40	1.13	1.11
	20190752	rs1250552	9×10^{-10}	G/A	0.53	0.56	1.12	1.09
	20190752	rs4819388	2×10^{-09}	T/C	0.72	0.70	1.14	1.10

Table S5: (continued)

Disease	PubMed ID	SNP	p-value	Alleles	$freq_{\text{study}}$	$freq_{\text{1000g}}$	$OR_{\text{uncorrected}}$	$OR_{\text{corrected}}$
	20190752	rs13314993	3×10^{-09}	T/G	0.46	0.47	1.13	1.09
	20190752	rs9792269	3×10^{-09}	G/A	0.76	0.71	1.14	1.10
	20190752	rs296547	4×10^{-09}	T/C	0.64	0.63	1.12	1.08
	20190752	rs11712165	8×10^{-09}	T/G	0.39	0.39	1.13	1.07
	20190752	rs12928822	3×10^{-08}	T/C	0.84	0.84	1.16	1.04
	20190752	rs1033180	6×10^{-08}	C/T	0.08	0.05	1.21	1.05
Colorectal cancer	20972440	rs4925386	2×10^{-10}	T/C	-	0.69	1.08	1.07
	20972440	rs11169552	2×10^{-10}	T/C	-	0.76	1.09	1.08
	20972440	rs6691170	1×10^{-09}	G/T	-	0.37	1.06	1.05
	20972440	rs10936599	3×10^{-08}	T/C	-	0.75	1.04	1.01
	18372905	rs6983267	7×10^{-11}	T/G	0.48	0.46	1.24	1.21
	18372901	rs4939827	8×10^{-28}	C/T	0.52	0.53	1.20	1.20
	18372901	rs3802842	6×10^{-10}	A/C	0.43	0.27	1.11	1.09
	21761138	rs4779584	2×10^{-08}	C/T	0.19	0.21	1.18	1.06
	19011631	rs961253	2×10^{-10}	C/A	0.36	0.38	1.12	1.10
	19011631	rs10411210	5×10^{-09}	T/C	0.90	0.91	1.15	1.10
	19011631	rs9929218	1×10^{-08}	G/A	0.29	0.28	1.10	1.05
Coronary artery disease	21378990	rs4977574	1×10^{-22}	A/G	0.46	0.49	1.29	1.29
	21378990	rs17114036	4×10^{-19}	G/A	0.91	0.89	1.17	1.17
	21378990	rs11556924	9×10^{-18}	T/C	0.62	0.61	1.09	1.09
	21378990	rs964184	1×10^{-17}	C/G	0.13	0.16	1.13	1.13
	21378990	rs579459	4×10^{-14}	T/C	0.21	0.21	1.10	1.10
	21378990	rs12190287	1×10^{-12}	G/C	0.62	0.60	1.08	1.08
	21378990	rs3825807	1×10^{-12}	G/A	0.57	0.60	1.08	1.08
	21378990	rs3798220	3×10^{-11}	T/C	0.02	0.02	1.51	1.46
	21378990	rs2895811	1×10^{-10}	T/C	0.43	0.43	1.07	1.06
	21378990	rs1746048	3×10^{-10}	T/C	0.87	0.86	1.09	1.08
	21378990	rs599839	3×10^{-10}	G/A	0.78	0.77	1.11	1.09
	21378990	rs9982601	4×10^{-10}	C/T	0.15	0.14	1.18	1.15
	21378990	rs12936587	4×10^{-10}	A/G	0.56	0.55	1.07	1.06
	21378990	rs1231206	9×10^{-10}	G/A	0.37	0.35	1.07	1.06
	21378990	rs6725887	1×10^{-09}	T/C	0.15	0.14	1.14	1.11
	21378990	rs1122608	1×10^{-09}	T/G	0.77	0.75	1.14	1.11
	21378990	rs12413409	1×10^{-09}	A/G	0.89	0.91	1.12	1.09
	21378990	rs12526453	1×10^{-09}	G/C	0.67	0.66	1.10	1.08
	21378990	rs4773144	4×10^{-09}	A/G	0.44	0.46	1.07	1.05
	21378990	rs17465637	1×10^{-08}	A/C	0.74	0.73	1.14	1.07
	21378990	rs46522	2×10^{-08}	C/T	0.53	0.55	1.06	1.02
	21378990	rs2306374	3×10^{-08}	T/C	0.18	0.15	1.12	1.03
	21606135	rs1412444	4×10^{-08}	C/T	0.32	0.36	1.10	1.03
	17634449	rs6922269	3×10^{-08}	G/A	0.25	0.30	1.23	1.06
Crohn disease	21102463	rs2076756	4×10^{-69}	A/G	0.26	0.24	1.53	1.53
	21102463	rs11209026	1×10^{-64}	A/G	0.93	0.93	2.66	2.66
	21102463	rs3792109	7×10^{-41}	G/A	0.53	0.52	1.34	1.34
	21102463	rs4077515	1×10^{-36}	C/T	0.41	0.42	1.18	1.18
	21102463	rs11742570	7×10^{-36}	T/C	0.61	0.60	1.33	1.33
	21102463	rs1250550	1×10^{-30}	A/C	0.67	0.69	1.19	1.19
	21102463	rs2413583	1×10^{-26}	T/C	0.83	0.83	1.23	1.23
	21102463	rs10761659	4×10^{-22}	A/G	0.54	0.56	1.23	1.23
	21102463	rs11564258	6×10^{-21}	G/A	0.03	0.04	1.74	1.74
	21102463	rs12521868	1×10^{-20}	G/T	0.42	0.38	1.23	1.23
	21102463	rs4409764	2×10^{-20}	G/T	0.49	0.47	1.22	1.22
	21102463	rs17293632	3×10^{-19}	C/T	0.23	0.21	1.12	1.12
	21102463	rs6651252	4×10^{-18}	C/T	0.87	0.89	1.23	1.23
	21102463	rs8005161	4×10^{-18}	C/T	0.12	0.13	1.23	1.23
	21102463	rs3197999	6×10^{-17}	G/A	0.30	0.28	1.22	1.22
	21102463	rs1819658	9×10^{-17}	T/C	0.77	0.80	1.19	1.19
	21102463	rs181359	5×10^{-16}	G/A	0.20	0.19	1.10	1.10
	21102463	rs4809330	3×10^{-15}	A/G	0.71	0.69	1.12	1.12
	21102463	rs10495903	2×10^{-14}	C/T	0.13	0.12	1.14	1.14
	21102463	rs3024505	2×10^{-14}	G/A	0.16	0.17	1.12	1.12
	21102463	rs3091315	2×10^{-13}	G/A	0.72	0.67	1.20	1.20
	21102463	rs7423615	3×10^{-13}	C/T	0.19	0.18	1.12	1.12
	21102463	rs12720356	1×10^{-12}	A/C	0.08	0.10	1.12	1.12

Table S5: (continued)

Disease	PubMed ID	SNP	p-value	Alleles	$freq_{\text{study}}$	$freq_{\text{1000g}}$	$OR_{\text{uncorrected}}$	$OR_{\text{corrected}}$
	21102463	rs359457	3×10^{-12}	C/T	0.57	0.58	1.08	1.08
	21102463	rs7702331	6×10^{-12}	G/A	0.60	0.65	1.12	1.11
	21102463	rs281379	7×10^{-12}	G/A	0.49	0.48	1.07	1.07
	21102463	rs713875	7×10^{-12}	G/C	0.47	0.44	1.08	1.08
	21102463	rs151181	2×10^{-11}	T/C	0.39	0.36	1.07	1.07
	21102463	rs212388	2×10^{-11}	T/C	0.39	0.45	1.10	1.09
	21102463	rs102275	2×10^{-11}	T/C	0.34	0.39	1.08	1.07
	21102463	rs780093	5×10^{-11}	C/T	0.42	0.42	1.15	1.14
	21102463	rs2549794	1×10^{-10}	T/C	0.41	0.42	1.05	1.04
	21102463	rs4902642	2×10^{-10}	A/G	0.58	0.58	1.07	1.06
	21102463	rs2062305	5×10^{-10}	A/G	0.35	0.47	1.10	1.08
	21102463	rs694739	6×10^{-10}	G/A	0.63	0.62	1.10	1.08
	21102463	rs13428812	9×10^{-10}	A/G	0.33	0.30	1.06	1.05
	21102463	rs11167764	2×10^{-09}	A/C	0.80	0.80	1.06	1.04
	21102463	rs6738825	4×10^{-09}	G/A	0.47	0.47	1.06	1.04
	21102463	rs1847472	5×10^{-09}	A/C	0.66	0.66	1.07	1.05
	21102463	rs13073817	7×10^{-09}	G/A	0.32	0.34	1.08	1.05
	21102463	rs17309827	7×10^{-09}	G/T	0.64	0.66	1.10	1.06
	21102463	rs10181042	7×10^{-09}	C/T	0.42	0.39	1.14	1.08
	21102463	rs2797685	7×10^{-09}	C/T	0.19	0.19	1.05	1.03
	21102463	rs736289	9×10^{-09}	C/T	0.61	0.62	1.06	1.03
	21102463	rs1998598	9×10^{-09}	A/G	0.30	0.29	1.04	1.02
	18587394	rs2542151	5×10^{-17}	T/G	0.15	0.14	1.35	1.35
	18587394	rs3764147	2×10^{-13}	A/G	0.22	0.27	1.25	1.24
	18587394	rs2301436	1×10^{-12}	C/T	0.46	0.45	1.21	1.20
	18587394	rs744166	7×10^{-12}	G/A	0.57	0.59	1.18	1.17
	18587394	rs11584383	1×10^{-11}	C/T	0.70	0.71	1.18	1.17
	18587394	rs7746082	2×10^{-10}	G/C	0.29	0.30	1.17	1.15
	18587394	rs762421	1×10^{-09}	A/G	0.39	0.42	1.13	1.10
	18587394	rs7927894	1×10^{-09}	C/T	0.39	0.36	1.16	1.12
	18587394	rs9286879	2×10^{-09}	A/G	0.24	0.24	1.19	1.14
	18587394	rs1551398	5×10^{-09}	G/A	0.62	0.61	1.08	1.05
	18587394	rs2872507	5×10^{-09}	G/A	0.47	0.46	1.12	1.08
	18587394	rs1456893	5×10^{-09}	G/A	0.68	0.68	1.20	1.13
	18587394	rs1736135	7×10^{-09}	C/T	0.57	0.60	1.18	1.11
	18587394	rs2476601	1×10^{-08}	A/G	0.90	0.93	1.31	1.16
	17554261	rs10801047	3×10^{-08}	T/A	0.08	0.08	1.47	1.11
Lung cancer	18978787	rs3117582	5×10^{-10}	T/G	-	0.08	1.24	1.20
	18978787	rs401681	8×10^{-09}	T/C	-	0.57	1.15	1.09
Melanoma	21983787	rs258322	3×10^{-27}	G/A	0.11	0.10	1.70	1.70
	21983787	rs1393350	2×10^{-13}	G/A	0.28	0.23	1.30	1.29
	21983787	rs13016963	9×10^{-10}	G/A	0.37	0.40	1.14	1.11
	21983787	rs45430	3×10^{-09}	C/T	0.61	0.64	1.14	1.10
	21983787	rs1801516	3×10^{-09}	A/G	0.87	0.82	1.19	1.13
	21983787	rs7023329	7×10^{-09}	G/A	0.51	0.55	1.20	1.12
	21983787	rs401681	3×10^{-08}	C/T	0.46	0.43	1.20	1.05
	21983785	rs7412746	9×10^{-11}	C/T	0.55	0.50	1.15	1.13
	19578364	rs2284063	2×10^{-09}	A/G	0.37	0.36	1.20	1.14
	18488026	rs910873	1×10^{-15}	G/A	0.09	0.05	1.75	1.74
	21706340	rs17119461	7×10^{-12}	T/C	0.04	0.01	8.40	7.52
Multiple sclerosis	21833088	rs7090512	5×10^{-20}	T/C	-	0.34	1.19	1.19
	21833088	rs669607	2×10^{-15}	A/C	-	0.51	1.13	1.13
	21833088	rs1738074	7×10^{-15}	T/C	-	0.54	1.13	1.13
	21833088	rs1077667	9×10^{-14}	T/C	-	0.76	1.16	1.16
	21833088	rs11154801	1×10^{-13}	C/A	-	0.39	1.13	1.13
	21833088	rs17066096	6×10^{-13}	A/G	-	0.20	1.14	1.14
	21833088	rs4902647	9×10^{-12}	T/C	-	0.54	1.11	1.10
	21833088	rs9282641	1×10^{-11}	A/G	-	0.90	1.21	1.20
	21833088	rs2546890	1×10^{-11}	G/A	-	0.48	1.11	1.10
	21833088	rs2248359	3×10^{-11}	T/C	-	0.59	1.12	1.11
	21833088	rs7595037	5×10^{-11}	C/T	-	0.58	1.11	1.10
	21833088	rs2119704	2×10^{-10}	A/C	-	0.93	1.22	1.19
	21833088	rs10201872	2×10^{-10}	C/T	-	0.17	1.14	1.12
	21833088	rs11581062	3×10^{-10}	A/G	-	0.26	1.12	1.10

Table S5: (continued)

Disease	PubMed ID	SNP	p-value	Alleles	$freq_{\text{study}}$	$freq_{\text{1000g}}$	$OR_{\text{uncorrected}}$	$OR_{\text{corrected}}$
	21833088	rs11129295	1×10^{-09}	C/T	-	0.41	1.11	1.09
	21833088	rs7238078	3×10^{-09}	G/T	-	0.77	1.12	1.08
	21833088	rs2303759	5×10^{-09}	T/G	-	0.25	1.11	1.07
	21833088	rs7923837	5×10^{-09}	A/G	-	0.63	1.10	1.06
	21833088	rs2283792	5×10^{-09}	T/G	-	0.51	1.10	1.06
	21833088	rs2019960	5×10^{-09}	T/C	-	0.22	1.12	1.08
	21833088	rs354033	5×10^{-09}	A/G	-	0.75	1.11	1.07
	21833088	rs802734	6×10^{-09}	G/A	-	0.72	1.10	1.06
	21833088	rs4410871	8×10^{-09}	T/C	-	0.68	1.11	1.06
	21833088	rs771767	9×10^{-09}	G/A	-	0.29	1.10	1.06
	21833088	rs10466829	1×10^{-08}	G/A	-	0.52	1.09	1.05
	21833088	rs874628	1×10^{-08}	G/A	-	0.72	1.11	1.06
	21833088	rs17174870	1×10^{-08}	T/C	-	0.73	1.11	1.06
	21833088	rs2300603	2×10^{-08}	C/T	-	0.74	1.11	1.04
	21833088	rs140522	2×10^{-08}	C/T	-	0.35	1.10	1.04
	21833088	rs12048904	4×10^{-08}	C/T	-	0.40	1.09	1.02
	22190364	rs2300747	6×10^{-09}	G/A	0.91	0.85	1.37	1.22
	22190364	rs2293152	4×10^{-08}	G/C	0.62	0.63	1.22	1.05
	19525953	rs1800693	2×10^{-11}	T/C	0.45	0.42	1.20	1.19
	19525953	rs17445836	4×10^{-09}	A/G	0.81	0.79	1.25	1.16
	19525953	rs17824933	4×10^{-09}	C/G	0.25	0.21	1.18	1.12
	19525955	rs703842	5×10^{-11}	G/A	0.67	0.68	1.23	1.21
	18997785	rs10492972	3×10^{-10}	T/C	0.27	0.34	1.34	1.28
Ovarian cancer	20852632	rs2072590	5×10^{-14}	C/A	-	0.33	1.16	1.16
	20852632	rs10088218	3×10^{-09}	A/G	-	0.89	1.19	1.13
	19648919	rs3814113	5×10^{-19}	C/T	0.68	0.67	1.22	1.22
Pancreatic cancer	19648918	rs505922	5×10^{-08}	T/C	0.35	0.35	1.20	1.05
	20101243	rs9543325	3×10^{-11}	T/C	0.37	0.38	1.26	1.24
	20101243	rs3790844	2×10^{-10}	G/A	0.76	0.75	1.30	1.26
Parkinson disease	21292315	rs356219	2×10^{-47}	A/G	0.39	0.40	1.29	1.29
	21292315	rs2942168	1×10^{-28}	A/G	0.78	0.75	1.27	1.27
	21292315	rs11724635	1×10^{-16}	C/A	0.56	0.58	1.15	1.15
	21292315	rs12817488	3×10^{-13}	G/A	0.41	0.47	1.14	1.14
	21292315	rs34372695	4×10^{-12}	C/T	0.03	0.02	1.47	1.44
	21292315	rs11711441	8×10^{-12}	A/G	0.86	0.81	1.19	1.18
	19915575	rs17115100	7×10^{-08}	T/G	0.91	0.91	1.25	1.05
	21738487	rs6599389	4×10^{-08}	G/A	0.07	0.08	1.31	1.07
	21738487	rs11868035	6×10^{-08}	A/G	0.69	0.65	1.18	1.04
	20711177	rs3129882	2×10^{-10}	A/G	0.40	0.45	1.26	1.22
Prostate cancer	19767754	rs1447295	2×10^{-19}	C/A	0.11	0.09	1.58	1.58
	19767754	rs16901979	3×10^{-14}	C/A	0.04	0.03	1.80	1.79
	19767754	rs11228565	7×10^{-12}	G/A	0.20	0.20	1.23	1.22
	19767754	rs8102476	2×10^{-11}	T/C	0.54	0.57	1.12	1.11
	19767754	rs10934853	3×10^{-10}	C/A	0.28	0.29	1.12	1.10
	19767754	rs445114	5×10^{-10}	C/T	0.64	0.64	1.14	1.11
	21743057	rs1859962	3×10^{-11}	T/G	-	0.48	1.27	1.25
	21743057	rs651164	2×10^{-10}	A/G	-	0.69	1.15	1.13
	21743057	rs902774	5×10^{-09}	G/A	0.17	0.14	1.17	1.11
	21743057	rs2292884	4×10^{-08}	A/G	0.25	0.20	1.14	1.03
	18264098	rs721048	8×10^{-09}	G/A	0.19	0.14	1.15	1.09
	18264097	rs10993994	9×10^{-29}	C/T	0.40	0.37	1.25	1.25
	18264097	rs2735839	2×10^{-18}	A/G	0.85	0.86	1.20	1.20
	18264097	rs9364554	6×10^{-10}	C/T	0.29	0.25	1.17	1.14
	18264097	rs6465657	1×10^{-09}	T/C	0.46	0.48	1.12	1.09
	18264097	rs2660753	3×10^{-08}	C/T	0.11	0.10	1.18	1.05
Schizophrenia	21926974	rs2021722	2×10^{-12}	T/C	0.78	0.82	1.15	1.14
	21926974	rs1625579	2×10^{-11}	G/T	0.80	0.78	1.12	1.11
	21926974	rs10503253	2×10^{-08}	C/A	0.19	0.18	1.16	1.06
	21926974	rs7914558	2×10^{-08}	A/G	0.59	0.56	1.22	1.08
	21926974	rs12966547	3×10^{-08}	A/G	0.58	0.61	1.40	1.10
	21926974	rs17662626	5×10^{-08}	G/A	0.91	0.93	1.20	1.05
	19571808	rs12807809	2×10^{-09}	C/T	0.83	0.84	1.15	1.11
	19571808	rs9960767	4×10^{-09}	A/C	0.06	0.06	1.23	1.15

Table S5: (continued)

Disease	PubMed ID	SNP	p-value	Alleles	$freq_{\text{study}}$	$freq_{\text{1000g}}$	$OR_{\text{uncorrected}}$	$OR_{\text{corrected}}$
Stroke	18991354	rs2200733	2×10^{-10}	C/T	0.11	0.14	1.26	1.22
Thyroid cancer	19198613	rs965513	2×10^{-27}	G/A	0.34	0.35	1.75	1.75
	19198613	rs944289	2×10^{-09}	C/T	0.57	0.57	1.37	1.26
Type 1 diabetes	19430480	rs10509540	1×10^{-28}	C/T	0.71	0.73	1.33	1.33
	19430480	rs5753037	3×10^{-16}	C/T	0.39	0.41	1.10	1.10
	19430480	rs7202877	3×10^{-15}	T/G	0.10	0.09	1.28	1.28
	19430480	rs9388489	4×10^{-13}	A/G	0.45	0.46	1.17	1.17
	19430480	rs2290400	6×10^{-13}	C/T	0.50	0.50	1.15	1.15
	19430480	rs1465788	2×10^{-12}	T/C	0.71	0.70	1.16	1.15
	19430480	rs7020673	5×10^{-12}	C/G	0.50	0.55	1.14	1.13
	19430480	rs2281808	1×10^{-11}	T/C	0.64	0.66	1.11	1.10
	19430480	rs4763879	2×10^{-11}	G/A	0.37	0.33	1.09	1.08
	19430480	rs425105	3×10^{-11}	C/T	0.84	0.83	1.16	1.15
	19430480	rs10517086	5×10^{-10}	G/A	0.30	0.27	1.09	1.07
	19430480	rs7221109	1×10^{-09}	T/C	0.65	0.63	1.05	1.04
	19430480	rs3024505	2×10^{-09}	A/G	0.83	0.83	1.19	1.14
	19430480	rs4900384	4×10^{-09}	A/G	0.29	0.27	1.09	1.06
	19430480	rs7804356	5×10^{-09}	C/T	0.76	0.80	1.14	1.09
	19430480	rs4948088	4×10^{-08}	A/C	0.95	0.97	1.30	1.07
	21980299	rs478222	4×10^{-09}	T/A	0.59	0.54	1.22	1.14
	21980299	rs924043	8×10^{-09}	T/C	0.85	0.85	1.35	1.19
	19966805	rs2304256	4×10^{-09}	A/C	0.71	0.71	1.16	1.11
	18978792	rs947474	4×10^{-09}	A/G	0.19	0.18	1.10	1.07
	18978792	rs229541	2×10^{-08}	G/A	0.43	0.41	1.11	1.04
	17554260	rs2476601	2×10^{-80}	G/A	0.09	0.07	1.98	1.98
	17554260	rs2292239	2×10^{-20}	G/T	0.34	0.31	1.28	1.28
	17554260	rs2542151	1×10^{-14}	T/G	0.16	0.14	1.30	1.30
	17554260	rs763361	1×10^{-08}	C/T	0.47	0.50	1.16	1.09
	18840781	rs9976767	2×10^{-08}	A/G	-	0.42	1.16	1.06
Type 2 diabetes	20581827	rs7903146	2×10^{-51}	C/T	-	0.32	1.40	1.40
	20581827	rs1552224	1×10^{-22}	C/A	-	0.87	1.14	1.14
	20581827	rs5015480	1×10^{-15}	T/C	-	0.56	1.18	1.18
	20581827	rs243021	3×10^{-15}	G/A	-	0.49	1.08	1.08
	20581827	rs1387153	8×10^{-15}	C/T	-	0.27	1.09	1.09
	20581827	rs231362	3×10^{-13}	A/G	-	0.51	1.08	1.08
	20581827	rs4457053	3×10^{-12}	A/G	-	0.30	1.08	1.08
	20581827	rs10965250	1×10^{-10}	A/G	-	0.84	1.20	1.18
	20581827	rs8042680	2×10^{-10}	C/A	-	0.35	1.07	1.06
	20581827	rs972283	2×10^{-10}	A/G	-	0.56	1.07	1.06
	20581827	rs896854	1×10^{-09}	C/T	-	0.46	1.06	1.05
	20581827	rs11634397	2×10^{-09}	A/G	-	0.65	1.06	1.04
	20581827	rs1470579	2×10^{-09}	A/C	-	0.29	1.14	1.10
	20581827	rs849134	3×10^{-09}	G/A	-	0.48	1.13	1.09
	20581827	rs3802177	1×10^{-08}	A/G	-	0.73	1.15	1.08
	20581827	rs11642841	3×10^{-08}	C/A	-	0.42	1.13	1.03
	18372903	rs12779790	1×10^{-10}	A/G	0.18	0.19	1.11	1.10
	18372903	rs7578597	1×10^{-09}	C/T	0.90	0.91	1.15	1.12
	18372903	rs4607103	1×10^{-08}	T/C	0.76	0.76	1.09	1.05
	18372903	rs10923931	4×10^{-08}	G/T	0.11	0.10	1.13	1.03
	20418489	rs7593730	4×10^{-08}	T/C	0.78	0.78	1.11	1.03
Ulcerative colitis	21297633	rs9268853	1×10^{-55}	C/T	0.66	0.66	1.40	1.40
	21297633	rs6426833	4×10^{-35}	G/A	0.54	0.51	1.30	1.30
	21297633	rs11209026	5×10^{-28}	A/G	0.94	0.93	1.74	1.74
	21297633	rs2836878	2×10^{-22}	A/G	0.74	0.73	1.25	1.25
	21297633	rs6871626	1×10^{-21}	C/A	0.33	0.36	1.17	1.17
	21297633	rs6584283	8×10^{-21}	C/T	0.47	0.47	1.21	1.21
	21297633	rs6017342	1×10^{-20}	A/C	0.54	0.55	1.20	1.20
	21297633	rs1801274	2×10^{-20}	G/A	0.51	0.52	1.21	1.21
	21297633	rs10781499	3×10^{-19}	G/A	0.41	0.42	1.12	1.12
	21297633	rs16940202	6×10^{-19}	T/C	0.18	0.18	1.15	1.15
	21297633	rs9822268	2×10^{-17}	G/A	0.30	0.28	1.21	1.21
	21297633	rs3024505	6×10^{-17}	G/A	0.16	0.17	1.25	1.25
	21297633	rs6920220	8×10^{-17}	G/A	0.21	0.15	1.14	1.14
	21297633	rs17085007	1×10^{-16}	T/C	0.18	0.17	1.16	1.16

Table S5: (continued)

Disease	PubMed ID	SNP	p-value	Alleles	$freq_{\text{study}}$	$freq_{\text{1000g}}$	$OR_{\text{uncorrected}}$	$OR_{\text{corrected}}$
	21297633	rs7134599	1×10^{-16}	G/A	0.39	0.35	1.19	1.19
	21297633	rs4510766	2×10^{-16}	G/A	0.56	0.56	1.20	1.20
	21297633	rs798502	3×10^{-15}	C/A	0.71	0.73	1.13	1.13
	21297633	rs7608910	2×10^{-14}	A/G	0.39	0.35	1.19	1.19
	21297633	rs7524102	2×10^{-13}	G/A	0.83	0.84	1.10	1.10
	21297633	rs1297265	7×10^{-13}	G/A	0.56	0.58	1.11	1.11
	21297633	rs2310173	3×10^{-12}	G/T	0.46	0.44	1.09	1.09
	21297633	rs941823	4×10^{-12}	T/C	0.76	0.77	1.12	1.11
	21297633	rs267939	6×10^{-12}	T/C	0.37	0.43	1.10	1.09
	21297633	rs2872507	5×10^{-11}	G/A	0.46	0.46	1.15	1.14
	21297633	rs2838519	6×10^{-11}	A/G	0.39	0.43	1.14	1.13
	21297633	rs4676406	8×10^{-11}	G/T	0.52	0.54	1.14	1.13
	21297633	rs907611	1×10^{-10}	G/A	0.32	0.34	1.08	1.07
	21297633	rs12261843	7×10^{-10}	T/G	0.29	0.28	1.07	1.06
	21297633	rs254560	1×10^{-09}	G/A	0.40	0.40	1.07	1.05
	21297633	rs734999	3×10^{-09}	T/C	0.52	0.53	1.05	1.03
	21297633	rs6451493	3×10^{-09}	G/T	0.61	0.60	1.08	1.06
	21297633	rs35675666	5×10^{-09}	T/G	0.83	0.86	1.08	1.05
	21297633	rs6911490	1×10^{-08}	C/T	0.21	0.22	1.08	1.04
	21297633	rs11739663	3×10^{-08}	C/T	0.77	0.78	1.15	1.04
	21297633	rs6499188	4×10^{-08}	G/A	0.75	0.80	1.14	1.03
	21297633	rs3194051	4×10^{-08}	A/G	0.27	0.29	1.07	1.02
	20228799	rs1317209	2×10^{-10}	G/A	-	0.15	1.17	1.15

Alleles are indicated as reference allele/variant allele. $freq_{\text{study}}$ and $freq_{\text{1000g}}$ provide the variant allele frequencies according to the NHGRI list and based on the 1000 Genomes data, respectively. $OR_{\text{uncorrected}}$ and $OR_{\text{corrected}}$ provide the original allelic odds ratio reported on the NHGRI list and its corresponding bias-corrected odds ratio.
