# Supplementary material for: Comparison of Family History and SNPs for Predicting Risk of Complex Disease
Source: PLoS Genet. 2012 Oct 11;8(10):e1002973. doi: 10.1371/journal.pgen.1002973 (PMC3469463; doi:10.1371/journal.pgen.1002973)
Supplement: Text S1 — Additional derivations. (PDF) [file pgen.1002973.s012.pdf]

Text S1: Additional derivations

Derivation of proportion of individuals exceeding a given post-test risk

The probability of obtaining a risk estimate, R, of t or greater is

$$P(R > t) = 1 - \Phi\left(\frac{T - \Phi^{-1}(1-t)\sqrt{1 - fh_L^2}}{\sqrt{fh_L^2}}\right),$$

where Φ is the cdf of the standard normal distribution, and $T = \Phi^{-1}(1-K)$. To see this, decompose the liability of an individual into measured and unmeasured components as $X = X_M + X_U$ where $X_M \sim \mathcal{N}(0, fh_L^2)$ and $X_U \sim \mathcal{N}(0, 1 - fh_L^2)$. Using the fact that the post-test risk is $R = P(X_U > T - X_M) = 1 - \Phi\left(\frac{T - X_M}{\sqrt{1 - fh_L^2}}\right)$, then $P(R > t) = P\left(X_M > T - \Phi^{-1}(1-t)\sqrt{1 - fh_L^2}\right) = 1 - \Phi\left(\frac{T - \Phi^{-1}(1-t)\sqrt{1-fh_L^2}}{\sqrt{fh_L^2}}\right)$.

Derivation of probability density function for the logarithm of the likelihood ratio

Let $R(\theta) = \frac{e^\theta K}{e^\theta K + 1 - K}$ be the post-test risk when the logarithm of the likelihood ratio is θ. Following the derivation above,

$$P(\log(\mathsf{LR}) < \theta) = \Phi\left(\frac{T - \Phi^{-1}(1 - R(\theta))\sqrt{1 - fh_L^2}}{\sqrt{fh_L^2}}\right)$$

so the density function is given by

$$\begin{aligned}\rho(\theta) &= \frac{\partial}{\partial\theta} P(\log(\mathsf{LR}) < \theta) \\ &= \phi\left(\frac{T - \Phi^{-1}(1 - R(\theta))\sqrt{1 - fh_L^2}}{\sqrt{fh_L^2}}\right) \cdot \sqrt{\frac{1 - fh_L^2}{fh_L^2}} \cdot \Phi^{-1\prime}(1 - R(\theta)) \cdot R'(\theta)\end{aligned}$$

where ϕ is the density function for a standard normal random variable. Applying the fact that $1 = \frac{d}{dx}x = \frac{d}{dx}\Phi(\Phi^{-1}(x)) = \Phi'(\Phi^{-1}(x))\Phi^{-1\prime}(x) = \phi(\Phi^{-1}(x))\Phi^{-1\prime}(x)$,

$$\begin{aligned}\rho(\theta) &= \phi\left(\frac{T - \Phi^{-1}(1 - R(\theta))\sqrt{1 - fh_L^2}}{\sqrt{fh_L^2}}\right) \cdot \sqrt{\frac{1 - fh_L^2}{fh_L^2}} \cdot \frac{1}{\phi(\Phi^{-1}(1 - R(\theta)))} \cdot \frac{e^\theta K(1-K)}{(e^\theta K + 1 - K)^2} \\ &= \frac{e^\theta K(1-K)}{(e^\theta K + 1 - K)^2}\sqrt{\frac{1 - fh_L^2}{fh_L^2}} \cdot e^z\end{aligned}$$

where

$$z = -\frac{1}{2}(\Phi^{-1}(1 - R(\theta)))^2 - \frac{\left(T - \Phi^{-1}(1 - R(\theta))\sqrt{1 - fh_L^2}\right)^2}{2fh_L^2}.$$

Including covariates in the liability-threshold model

In the main text, we discussed various approaches to handling sex-dependence of phenotypes. Here, we describe an approach which explicitly models sex as a covariate in the model. We note that this approach extends easily to arbitrary discrete covariates.

Consider a modified liability threshold model in which an individual's disease liability is decomposed into additive genetic, environmental, and sex contributions, $X_i = G_i + E_i + S_i$. As before, we assume that $G_1, \ldots, G_m$ are sampled from a multivariate normal distribution with zero mean and covariance matrix $h_L^2 C$.

This time, however, we additionally model sex contributions to liability for each individual in the pedigree as being independently sampled from a Bernoulli distribution. Notationally, we refer to the two outcomes of the Bernoulli distribution as s_1 and s_2 and their corresponding probabilities as p_1 and p_2 (where $p_1 + p_2 = 1$); without loss of generality, we assume that $\sum_j p_j s_j = 0$. Letting h_S^2 denote the total variance in liability arising from sex effects, and assuming that $E_1, \ldots, E_m$ are each independently sampled from a zero-mean normal distribution with variance $1 - h_L^2 - h_S^2$, it follows that $E[X_i] = E[G_i] + E[E_i] + E[S_i] = 0$ and $\mathrm{Var}[X_i] = \mathrm{Var}[G_i] + \mathrm{Var}[E_i] + \mathrm{Var}[S_i] = h_L^2 + (1 - h_L^2 - h_S^2) + h_S^2 = 1$.

Let K_1 and K_2 denote the sex-specific disease frequencies for males and females, respectively. The liability contributions s_j for each sex can be determined from the sex-specific frequencies K_j by noting that within any sex stratum, the genetic and environmental contributions to liability are normally distributed, i.e., $X|S = s_j \sim \mathcal{N}(s_j, 1 - h_S^2)$. If T denotes the threshold on total liability beyond which the disease manifests, then $\frac{T - s_j}{\sqrt{1 - h_S^2}} = \Phi^{-1}(1 - K_j)$ in order for the proportion of cases in the jth stratum to be K_j. Solving for s_j, we have $s_j = T - \Phi^{-1}(1 - K_j)\sqrt{1 - h_S^2}$. To find T and h_S^2, observe that

$$0 = \sum_j p_j s_j = \sum_j p_j \left[T - \Phi^{-1}(1 - K_j)\sqrt{1 - h_S^2} \right]$$

$$h_S^2 = \sum_j p_j s_j^2 = \sum_j p_j \left[T - \Phi^{-1}(1 - K_j)\sqrt{1 - h_S^2} \right]^2.$$

From the first equation, it follows that $T = \sum_j p_j \Phi^{-1}(1 - K_j)\sqrt{1 - h_S^2}$. Substituting into the second equation,

$$\begin{aligned} h_S^2 &= \sum_j p_j \left[\left(\sum_{j'} p_{j'} \Phi^{-1}(1 - K_{j'}) - \Phi^{-1}(1 - K_j) \right) \sqrt{1 - h_S^2} \right]^2 \\ &= (1 - h_S^2) \left[\sum_j p_j \left(\sum_{j'} p_{j'} \Phi^{-1}(1 - K_{j'}) - \Phi^{-1}(1 - K_j) \right)^2 \right] \\ &= (1 - h_S^2) \left[\left(\sum_j p_j (\Phi^{-1}(1 - K_j))^2 \right) - \left(\sum_j p_j \Phi^{-1}(1 - K_j) \right)^2 \right] \\ &= (1 - h_S^2)\, \mathrm{Var}\left[\Phi^{-1}(1 - K_j) \right]. \end{aligned}$$

Letting $z = \mathrm{Var}\left[\Phi^{-1}(1 - K_j)\right]$, it follows that $h_S^2 = \frac{z}{1+z}$; values for T and each of the s_j follow immediately.

Evaluating the performance of a family history-based classifier that accounts for sex can be done in a manner analogous to what has been described already; we simply modify all estimates of disease risk $P(D_1 | D_2, \ldots, D_m)$ by conditioning on the known sex of each individual in the pedigree, i.e., $P(D_1 | D_2, \ldots, D_m, S_1 = s_1, \ldots, S_m = s_m)$.
