# Supplementary material for: Comparison of Family History and SNPs for Predicting Risk of Complex Disease
Source: PLoS Genet. 2012 Oct 11;8(10):e1002973. doi: 10.1371/journal.pgen.1002973 (PMC3469463; doi:10.1371/journal.pgen.1002973)
Supplement: Text S2 — References for disease frequency and heritability. (PDF) [file pgen.1002973.s013.pdf]

Text S2: References for disease frequency and heritability

Citations to papers providing lifetime morbid risks and heritabilities, and proportions of heritability explained are provided in Tables S6 and S7. Where multiple values for heritability are shown in Table S7, we used an inverse variance weighted average to obtain the value shown in Table 2.

For age-related macular degeneration (AMD), celiac disease, Crohn disease, and ulcerative colitis, the values of lifetime morbid risk were estimated based on available incidence data. Incidence rates for late AMD (i.e., geographic atrophy or neovascular AMD) were based on the Rotterdam Study [1]. In the case of celiac disease, a constant incidence rate of 9.1 cases per 100,000 individuals per year was assumed [2]. Incidence numbers for Crohn disease and ulcerative colitis were taken from a recent study of inflammatory bowel disease rates in a Northern California managed care organization [3]. To model competing risks, we used overall United States mortality rates [4].

Let i_t and m_t denote the incidence and mortality rates at age t, let p_t denote the probability of developing the disease before age t, and let q_t denote the probability of surviving to age t without developing the disease or dying from other causes. Then,

$$p_t = p_{t-1} + q_{t-1} \cdot \frac{i_t}{i_t + m_t} \cdot \exp(-i_t - m_t)$$
$$q_t = q_{t-1} \cdot (1 - \exp(-i_t - m_t)), 1$$

where $p_0 = 0$ and $q_0 = 1$. Then, p_{90} is an estimate of the lifetime risk of developing the disease to age 90.

References

[1] van Leeuwen R, Klaver CC, Vingerling JR, Hofman A, de Jong PT (2003) The risk and natural course of age-related maculopathy: follow-up at 6 1/2 years in the Rotterdam study. Arch Ophthalmol 121: 519–526.

[2] Murray JA, Van Dyke C, Plevak MF, Dierkhising RA, Zinsmeister AR, et al. (2003) Trends in the identification and clinical features of celiac disease in a North American community, 1950-2001. Clin Gastroenterol Hepatol 1: 19–27.

[3] Herrinton LJ, Liu L, Lewis JD, Griffin PM, Allison J (2008) Incidence and prevalence of inflammatory bowel disease in a Northern California managed care organization, 1996-2002. Am J Gastroenterol 103: 1998–2006.

[4] Miniño AM, Murphy SL, Xu J, Kochanek KD (2011) Deaths: Final Data for 2008. National Vital Statistics Reports 56.

[5] Seshadri S, Beiser A, Kelly-Hayes M, Kase CS, Au R, et al. (2006) The lifetime risk of stroke: estimates from the Framingham Study. Stroke 37: 345–350.

[6] Lloyd-Jones DM, Wang TJ, Leip EP, Larson MG, Levy D, et al. (2004) Lifetime risk for development of atrial fibrillation: the Framingham Heart Study. Circulation 110: 1042–1046.

[7] Kessler RC, Berglund P, Demler O, Jin R, Merikangas KR, et al. (2005) Lifetime prevalence and age-of-onset distributions of DSM-IV disorders in the National Comorbidity Survey Replication. Arch Gen Psychiatry 62: 593–602.

[8] Howlader N, Noone AM, Krapcho M, Neyman N, Aminou R, et al. (2011). SEER Cancer Statistics Review, 1975-2008, National Cancer Institute. Bethesda, MD, http://seer.cancer.gov/csr/1975_2008/, based on November 2010 SEER data submission, posted to the SEER web site, 2011.

[9] Lloyd-Jones DM, Larson MG, Beiser A, Levy D (1999) Lifetime risk of developing coronary heart disease. Lancet 353: 89–92.

[10] Alonso A, Hernan MA (2008) Temporal trends in the incidence of multiple sclerosis: a systematic review. Neurology 71: 129–135.

[11] Elbaz A, Bower JH, Maraganore DM, McDonnell SK, Peterson BJ, et al. (2002) Risk tables for parkinsonism and Parkinson's disease. J Clin Epidemiol 55: 25–31.

[12] Saha S, Chant D, Welham J, McGrath J (2005) A systematic review of the prevalence of schizophrenia. PLoS Med 2: e141.

[13] Narayan KM, Boyle JP, Thompson TJ, Sorensen SW, Williamson DF (2003) Lifetime risk for diabetes mellitus in the United States. JAMA 290: 1884–1890.

[14] Seddon JM, Cote J, Page WF, Aggen SH, Neale MC (2005) The US twin study of age-related macular degeneration: relative roles of genetic and environmental influences. Arch Ophthalmol 123: 321–327.

[15] Gatz M, Reynolds CA, Fratiglioni L, Johansson B, Mortimer JA, et al. (2006) Role of genes and environments for explaining Alzheimer disease. Arch Gen Psychiatry 63: 168–174.

[16] Christophersen IE, Ravn LS, Budtz-Joergensen E, Skytthe A, Haunsoe S, et al. (2009) Familial aggregation of atrial fibrillation: a study in Danish twins. Circ Arrhythm Electrophysiol 2: 378–383.

[17] McGuffin P, Rijsdijk F, Andrew M, Sham P, Katz R, et al. (2003) The heritability of bipolar affective disorder and the genetic relationship to unipolar depression. Arch Gen Psychiatry 60: 497–502.

[18] Lichtenstein P, Yip BH, Bjork C, Pawitan Y, Cannon TD, et al. (2009) Common genetic determinants of schizophrenia and bipolar disorder in Swedish families: a population-based study. Lancet 373: 234–239.

[19] Lichtenstein P, Holm NV, Verkasalo PK, Iliadou A, Kaprio J, et al. (2000) Environmental and heritable factors in the causation of cancer–analyses of cohorts of twins from Sweden, Denmark, and Finland. N Engl J Med 343: 78–85.

[20] Czene K, Lichtenstein P, Hemminki K (2002) Environmental and heritable causes of cancer among 9.6 million individuals in the Swedish Family-Cancer Database. Int J Cancer 99: 260–266.

[21] Nistico L, Fagnani C, Coto I, Percopo S, Cotichini R, et al. (2006) Concordance, disease progression, and heritability of coeliac disease in Italian twins. Gut 55: 803–808.

[22] Fischer M, Broeckel U, Holmer S, Baessler A, Hengstenberg C, et al. (2005) Distinct heritable patterns of angiographic coronary artery disease in families with myocardial infarction. Circulation 111: 855–862.

[23] Tysk C, Lindberg E, Jarnerot G, Floderus-Myrhed B (1988) Ulcerative colitis and Crohn's disease in an unselected population of monozygotic and dizygotic twins. A study of heritability and the influence of smoking. Gut 29: 990–996.

[24] Sofaer J (1993) Crohn's disease: the genetic contribution. Gut 34: 869–871.

[25] Hawkes CH, Macgregor AJ (2009) Twin studies and the heritability of MS: a conclusion. Mult Scler 15: 661–667.

[26] Tanner CM, Ottman R, Goldman SM, Ellenberg J, Chan P, et al. (1999) Parkinson disease in twins: an etiologic study. JAMA 281: 341–346.

[27] Sullivan PF, Kendler KS, Neale MC (2003) Schizophrenia as a complex trait: evidence from a meta-analysis of twin studies. Arch Gen Psychiatry 60: 1187–1192.

[28] Bak S, Gaist D, Sindrup SH, Skytthe A, Christensen K (2002) Genetic liability in stroke: a long-term follow-up study of Danish twins. Stroke 33: 769–774.

[29] Hyttinen V, Kaprio J, Kinnunen L, Koskenvuo M, Tuomilehto J (2003) Genetic liability of type 1 diabetes and the onset age among 22,650 young Finnish twin pairs: a nationwide follow-up study. Diabetes 52: 1052–1055.

[30] Kyvik KO, Green A, Beck-Nielsen H (1995) Concordance rates of insulin dependent diabetes mellitus: a population based study of young Danish twins. BMJ 311: 913–917.

[31] Poulsen P, Kyvik KO, Vaag A, Beck-Nielsen H (1999) Heritability of type II (non-insulin-dependent) diabetes mellitus and abnormal glucose tolerance–a population-based twin study. Diabetologia 42: 139–145.

[32] Almgren P, Lehtovirta M, Isomaa B, Sarelin L, Taskinen MR, et al. (2011) Heritability and familiality of type 2 diabetes and related quantitative traits in the Botnia Study. Diabetologia 54: 2811–2819.

[33] Hunt KJ, Lehman DM, Arya R, Fowler S, Leach RJ, et al. (2005) Genome-wide linkage analyses of type 2 diabetes in Mexican Americans: the San Antonio Family Diabetes/Gallbladder Study. Diabetes 54: 2655–2662.

Table S6: **Disease frequency statistics.**

Disease	K	Notes
Age-related macular degeneration	0.0472	Estimated using cumulative incidence adjusted for competing risks [1, 4]
Alzheimer disease	0.132	Table 3, sex-averaged remaining lifetime risk at age 55 [5]
Atrial fibrillation	0.245	Table 2, sex-averaged remaining lifetime risk at age 40 [6]
Bipolar disorder	0.051	Table 3, projected lifetime risk to age 75 [7]
Bladder cancer	0.0241	Table 1.14, lifetime risk in both sexes [8]
Breast cancer	0.1229	Table 1.16, lifetime risk in females [8]
Celiac disease	0.0070	Estimated using cumulative incidence adjusted for competing risks [2, 4]
Colorectal cancer	0.0508	Table 1.14, lifetime risk in both sexes [8]
Coronary artery disease	0.402	Table 3, sex-averaged remaining lifetime risk at age 40 [9]
Crohn disease	0.0051	Estimated using cumulative incidence adjusted for competing risks [3, 4]
Lung cancer	0.0694	Table 1.14, lifetime risk in both sexes [8]
Melanoma	0.0197	Table 1.14, lifetime risk in both sexes [8]
Multiple sclerosis	0.020	Table 1, lifetime risk in both sexes [10]
Ovarian cancer	0.0140	Table 1.16, lifetime risk in females [8]
Pancreatic cancer	0.0145	Table 1.14, lifetime risk in both sexes [8]
Parkinson disease	0.016	Table 3, lifetime risk in both sexes [11]
Prostate cancer	0.1648	Table 1.15, lifetime risk in males [8]
Schizophrenia	0.0072	Lifetime morbid risk in both sexes [12]
Stroke	0.190	Table 1, sex-averaged remaining lifetime risk at age 55 [5]
Thyroid cancer	0.0097	Table 1.14, lifetime risk in both sexes [8]
Type 1 diabetes	0.018	Tables 1 and 2, sex-averaged lifetime risk of diabetes, multiplied by estimated proportion (5%) of type 1 diabetes [13]
Type 2 diabetes	0.339	Tables 1 and 2, sex-averaged lifetime risk of diabetes, multiplied by estimated proportion (95%) of type 2 diabetes [13]
Ulcerative colitis	0.0091	Estimated using cumulative incidence adjusted for competing risks [3, 4]

Values provided are used here as estimates of lifetime morbid risk.

Table S7: **Disease heritability statistics.**

Disease	h^2_L	Notes
Age-related macular degeneration	0.71 (0.18-0.88)	Estimated using twin pairs from the National Academy of Sciences/National Research Council World War II Veteran Twins Registry [14]
Alzheimer disease	0.79 (0.67-0.88)	Estimated using twin pairs from the Swedish Twin Registry [15]
Atrial fibrillation	0.62 (0.55-0.68)	Estimated using twin pairs from the Danish Twin Register [16]
Bipolar disorder	0.85 (0.73-0.93)	Estimated using twin pairs from the Maudley Twin Register [17]
	0.586 (0.564-0.618)	Estimated using families from Sweden [18]
Bladder cancer	0.31 (0.00-0.45)	Estimated using twin pairs from Swedish, Danish, and Finnish twin registries [19]
	0.07 (0.02-0.11)	Estimated using families from the Swedish Family-Cancer Database [20]
Breast cancer	0.27 (0.04-0.41)	Estimated using twin pairs from Swedish, Danish, and Finnish twin registries [19]
	0.25 (0.23-0.27)	Estimated using families from the Swedish Family-Cancer Database [20]
Celiac disease	0.57 (0.32-0.93)	Estimated assuming population prevalence of 1/1000 using twin pairs from the Italian Twin Registry [21]
	0.87 (0.49-1.00)	Estimated assuming population prevalence of 1/91 using twin pairs from the Italian Twin Registry [21]
Colorectal cancer	0.35 (0.10-0.48)	Estimated using twin pairs in Swedish, Danish, and Finnish twin registries [19]
	0.13 (0.12-0.18)	Colon cancer only; estimated using families in Swedish Family-Cancer Database [20]
	0.12 (0.08-0.13)	Rectal cancer only; estimated using families in Swedish Family-Cancer Database [20]
Coronary artery disease	0.49 (0.25-0.73)	Estimated using families from Germany [22]
Crohn disease	1.00 (0.34-1.00)	Estimated using twin pairs from the Swedish Twin Registry [23]
	0.55 (0.50-0.60)	Estimate from review of family studies [24]
Lung cancer	0.26 (0.00-0.49)	Estimated using twin pairs in Swedish, Danish, and Finnish twin registries [19]
	0.08 (0.05-0.09)	Estimated using families from the Swedish Family-Cancer Database [20]
Melanoma	0.21 (0.12-0.23)	Estimated using families from the Swedish Family-Cancer Database [20]
Multiple sclerosis	0.51 (0.25-0.76)	Median estimate from review of twin studies [25]
Ovarian cancer	0.22 (0.00-0.41)	Estimated using twin pairs in Swedish, Danish, and Finnish twin registries [19]
Pancreatic cancer	0.36 (0.00-0.53)	Estimated using twin pairs in Swedish, Danish, and Finnish twin registries [19]
Parkinson disease	0.274 (0.000-0.708)	Estimated using twin pairs from the National Academy of Sciences/National Research Council World War II Veteran Twins Registry [26]
Prostate cancer	0.42 (0.29-0.50)	Estimated using twin pairs in Swedish, Danish, and Finnish twin registries [19]
Schizophrenia	0.81 (0.73-0.90)	Estimated from meta-analysis of twin studies [27]
	0.643 (0.617-0.675)	Estimated using families from Sweden [18]
Stroke	0.17 (0.00-0.42)	Estimated using twin pairs from the Danish Twin Register [28]
Thyroid cancer	0.53 (0.52-0.53)	Estimated using families from the Swedish Family-Cancer Database [20]
Type 1 diabetes	0.88 (0.78-0.94)	Estimated using twin pairs from the Finnish Twin Cohort [29]
	0.72 (0.31-1.00)	Estimated using twin pairs from the Danish Twin Register [30]
Type 2 diabetes	0.26 (0.00-0.85)	Estimated using twin pairs from the Danish Twin Register [31]
	0.25 (0.20-0.30)	Estimated using families from the Botnia Study in Finland [32]
	0.56 (0.45-0.67)	Estimated using genotypic data for Mexican-American families from the San Antonio Family Diabetes/Gallbladder Study [33]
Ulcerative colitis	0.53 (0.24-0.82)	Estimated using monozygotic twin pairs from Swedish Twin Registry[23]

Values shown correspond to estimates of heritability from the literature, along with their 95% confidence intervals. For some disease, more than one estimate is provided, and as seen in the table, heritability estimates for a given disease can differ widely between studies. In such situations, we used an inverse variance weighted average to obtain the value shown in Table 2.
